# CellTrap: an instrument-free microfluidic platform for cell–cell interactions at stochastically generated effector-to-target ratios

**DOI:** 10.1039/d6ra02345b

**Published:** 2026-04-10

**Authors:** Muhammad Zia Ullah Khan, Morteza Hasanzadeh Kafshgari, Ali Bashiri Dezfouli, Oliver Hayden, Gabriele Multhoff, Ghulam Destgeer

**Affiliations:** a Control and Manipulation of Microscale Living Objects, Center for Translational Cancer Research (TranslaTUM), Munich Institute of Biomedical Engineering (MIBE), Munich Institute of Integrated Materials, Energy and Process Engineering (MEP), Department of Electrical Engineering, School of Computation, Information and Technology (CIT), Technical University of Munich (TUM) Einsteinstraße 25 Munich 81675 Germany ghulam.destgeer@tum.de; b Heinz-Nixdorf-Chair of Biomedical Electronics, TranslaTUM, MIBE, School of Computation, Information and Technology, Technical University of Munich Einsteinstraße 25 81675 Munich Germany; c Experimental Radiation Oncology and Radiobiology, TranslaTUM, School of Medicine, Technical University of Munich Einsteinstraße 25 81675 Munich Germany; d Department of Otolaryngology, Head and Neck Surgery, TUM School of Medicine and Health, Technical University of Munich 81675 Munich Germany

## Abstract

Immune–cancer cell interactions play a central role in understanding antitumor responses and evaluating immunotherapies. However, long-term, single-cell-level analysis of these interactions remains challenging. To address this, we developed CellTrap, an instrument-free, perfusion-capable microfluidic device featuring 1024 parallel traps. Each trap is equipped with a filter constriction to stably retain cells under hydrostatic flow, sustain continuous medium perfusion, and minimize trap-to-trap crosstalk. By intentionally leveraging stochastic Poisson loading, a single seeding step simultaneously generates perfectly matched internal controls alongside variable effector-to-target (E : T) ratios across the array. Device characterization using 10 µm fluorescent beads and cells validated the predictable trap occupancy governed by Poisson statistics. Initial proof-of-concept experiments using primary human PBMCs against GFP-expressing U87 (U87^GFP^) glioblastoma cells successfully demonstrated targeted, immune-mediated cytotoxicity over 14 hours. To decouple donor-derived biological heterogeneity from technical validation, we subsequently employed IL-2-stimulated Natural Killer cells (NK92^IL2^) as a standardized effector population against U87^GFP^, K562 leukemia, and LS174T adenocarcinoma targets. Continuous time-lapse imaging seamlessly linked early transient intracellular calcium fluxes (indicating target engagement) to distinct, contact-dependent cytotoxic outcomes. Our data demonstrate that increasing E : T ratios consistently enhances immune-mediated target lysis, highlighting the platform's robust utility for dissecting complex immune–cancer dynamics and guiding the development of personalized immunotherapies.

## Abstract 2

Our instrument-free microfluidic platform enables long-term, single-cell analysis of immune-mediated tumor killing. The device allows medium exchange, minimizes cross-talk, and captures activation dynamics and ratio-dependent cytotoxicity.

## Graphical abstract

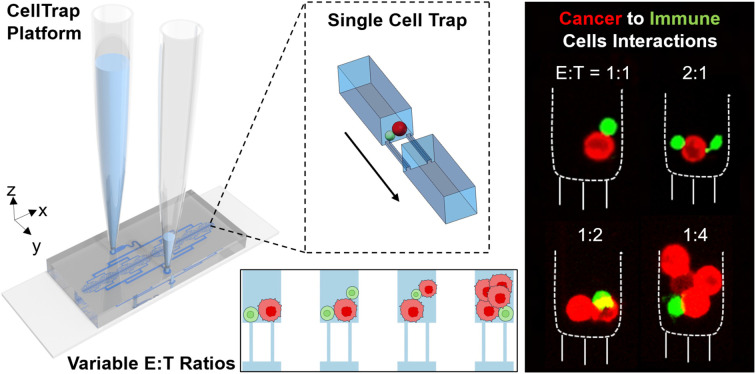

## 1. Introduction

Cell-to-cell interactions are crucial for understanding the complexity of immune responses. They underpin our understanding of tumor survival mechanisms,^1^ drive the development of advanced immunotherapies such as CAR T-cell and CAR NK-cell therapies, enable personalized treatment strategies,^2^ and inform efforts to prevent metastasis.^3^

To investigate these intricate immune dynamics, researchers employ a range of *in vivo*, *ex vivo*, and *in vitro* methodologies, each offering distinct strengths and facing specific limitations.^4^*In vivo* models provide the most biologically comprehensive insights but are often constrained by ethical considerations, technological challenges, extended timelines, and high costs. *Ex vivo* approaches, which rely on biopsies or intact tissues, enable the direct examination of human or animal samples; however, they are limited by the restricted availability of patient material and issues such as tissue degradation over time.

In contrast, *in vitro* systems offer accessibility and scalability, making them a mainstay in immunological research. Yet, conventional *in vitro* platforms, such as Petri dishes and well plates, lack fine control over the cellular microenvironment. As a result, they tend to average out cellular responses, obscuring critical single-cell interactions within heterogeneous populations. Moreover, their dependence on large cell quantities hinders the study of rare primary cells or genetically engineered immune subsets.

Microfluidics-based platforms have emerged as a promising alternative, addressing many of these limitations by enabling high-resolution analysis^5^ and real-time observation of cell behavior and interactions.^6,7^ These systems offer precise control over the microenvironment, *i.e.*, regulating chemical gradients, cell numbers, and interaction timeframes, while requiring only minimal sample and reagent volumes.^8,9^ Such features make microfluidic technologies highly attractive for applications in immunotherapy^10^ and personalized medicine.^11^

A variety of microfluidic architectures have been developed to study cell–cell interactions, encompassing droplet-based systems,^12–31^ static microwells,^32–35^ and open array-based cell traps.^12,14,36–48^ As outlined in Table S1, these microfluidic platforms can be broadly categorized by their cell-pairing strategies into deterministic and stochastic approaches. Deterministic methods, which can be further categorized into passive and active mechanisms, enable controlled cell pairing and achieve high one-to-one pairing efficiencies. Passive deterministic systems typically rely on size-based cell trapping or inertial flow focusing and demand precisely engineered microstructures to ensure proper alignment and capture. Active deterministic approaches, on the other hand, employ external forces such as electric, magnetic, acoustic, or optical fields to manipulate cells. While highly effective, these systems often require expensive instrumentation, specialized expertise, and substantial physical space. These factors hinder their widespread use in routine biological research. In contrast, stochastic pairing, governed by Poisson statistics, offers a simple and scalable alternative across both droplet and array-based formats. It accommodates a broad range of cell sizes and simplifies device design, albeit at the cost of lower one-to-one pairing efficiency. Despite these trade-offs, both deterministic and stochastic platforms have provided critical insights into immune–cancer cell interactions, enabling analyses such as time-lapse imaging, calcium flux monitoring, cytokine release assays, and cytotoxicity measurements across a diverse range of E : T ratios.

Beyond pairing mechanisms, each of these architectures entails specific fluidic and operational trade-offs (systematically compared in Table S1). Open array-based and microwell systems facilitate continuous perfusion, sequential staining, and cell adhesion; however, they are prone to high biochemical crosstalk between adjacent wells or traps due to a shared bulk medium, and typically require high cell concentrations to achieve sufficient occupancy. Droplet microfluidic systems, by contrast, utilize oil emulsions to effectively prevent crosstalk but inherently preclude continuous medium exchange and cell adhesion. While recent advancements in passive deterministic droplet encapsulation have successfully improved 1 : 1 pairing efficiencies beyond standard Poisson limits^22^ they still involve technically complex setups, relying on sealed emulsions, stabilization devices for imaging, and pre-mixing of staining reagents, which can introduce cytotoxic effects. Moreover, both deterministic array-based systems and continuous droplet generators often rely on expensive syringe pumps, which significantly increases system complexity and cost. Together, these limitations highlight the need for a microfluidic platform that leverages the strengths of existing systems while mitigating their drawbacks. Specifically, this requires a device that enables rapid and controlled cell seeding, supports sequential medium exchange with low crosstalk, operates entirely instrument-free, and accommodates small sample volumes for rare or engineered cell populations.

**Fig. 1:**
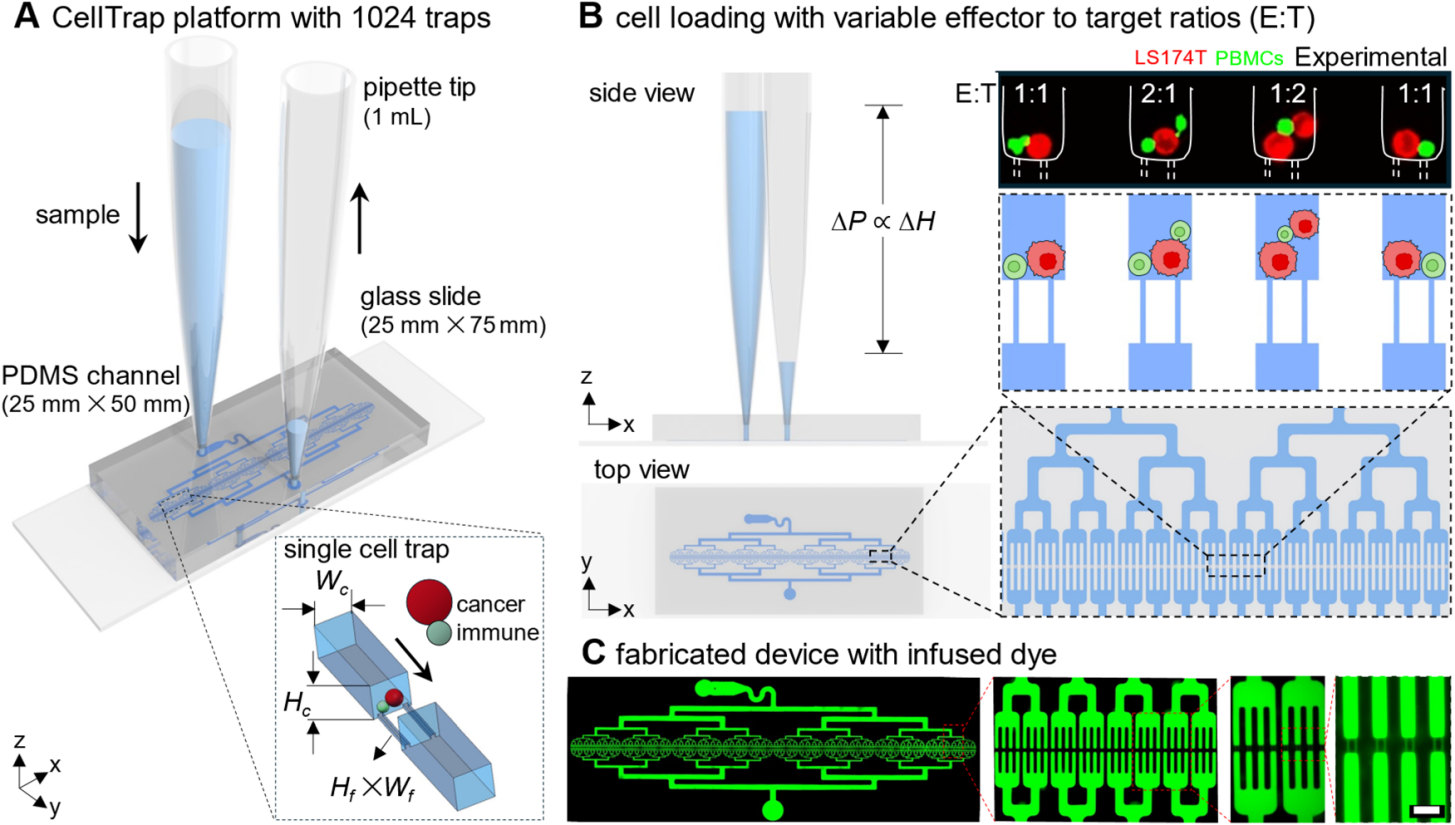
An instrument-free operation of the CellTrap platform. (A) A PDMS microfluidic channel containing 1024 traps is bonded to a glass slide. Sample loading is driven by hydrostatic pressure generated by the fluid column at the inlet pipette tip. The main channel height (*H*_c_) and width (*W*_c_) are larger than the cell diameter (*D*_c_) to allow uninterrupted cell flow, whereas the filter height (*H*_f_) and width (*W*_f_) at each trap are smaller than *D*_c_ to ensure cell capture. (B) The hydrostatic pressure gradient (Δ*P*) is proportional to the height difference between the inlet and outlet free surfaces. Cells are trapped at variable E : T ratios, *e.g.*, 1 : 1, 2 : 1, 1 : 2, and 1 : 1. (C) Fabricated device infused with Rhodamine B dye to highlight the channel network. Scale bar: 50 µm.

To meet these requirements, we have developed CellTrap, an instrument-free, perfusion-capable polydimethylsiloxane (PDMS)-based microfluidic platform, featuring 1024 parallel-arranged traps in a 1D array, specifically designed for long-term co-incubation and real-time visualization of hundreds of cell–cell interactions (Fig. 1). The gas-permeable PDMS channel enables sustained culture and live-cell imaging using standard microscopes equipped with environmental control. The platform supports rapid, instrument-free sample loading through hydrostatic pressure-driven flow, ensuring robust operation and enabling efficient medium exchange with minimal crosstalk (Fig. 1A).

Crucially, while deterministic active platforms successfully maximize 1 : 1 pairing efficiencies, their reliance on complex instrumentation limits their accessibility. Conversely, the CellTrap platform intentionally leverages the stochastic nature of Poisson loading. Rather than viewing non-1 : 1 traps as inefficient, this approach exploits the natural statistical distribution to simultaneously generate perfectly matched on-chip controls (target-only or effector-only traps) alongside a spectrum of variable E : T interaction ratios (*e.g.*, 1 : 1, 1 : 2, 2 : 1) within a single, user-friendly assay.

The channel geometry is optimized for selective trapping: the main channel height (*H*_c_) and width (*W*_c_) exceed the average cell diameter (*D*_c_) to facilitate smooth flow, while the filter height (*H*_f_) and width (*W*_f_) at the trap are smaller than *D*_c_ to capture cells effectively. For example, a single cancer cell (red) paired with an engineered immune cell (green) can be co-incubated for several days, allowing quantitative time-lapse analysis of immune efficacy. Each trap is capable of stochastically capturing two distinct cells, generating controlled pairings across a natural spectrum of E : T ratios (Fig. 1B).

The CellTrap platform is fabricated with eight bifurcation stages highlighted by the infusion of channels with a fluorescent dye (Fig. 1C). At the final stage of bifurcation, the main channel splits into four parallel daughter channels that terminate at the cell trap, each with two to three filter channels to capture cancer and immune cells. We have characterized our CellTrap platform for stochastic loading of cells. As a proof of concept, we first performed cytotoxicity assays to investigate interactions between U87^GFP^ cells and peripheral blood mononuclear cells (PBMCs). Secondly, we performed calcium and cytotoxicity assays to investigate the interactions of three cancer cell lines, *i.e.*, glioblastoma U87^GFP^, chronic myelogenous leukemia K562, and adenocarcinoma LS174T, against NK92^IL2^ immune cells at variable E : T ratios. We have selected these cancer cell types for their diverse range and levels of ligand expression that activate or inhibit the response of NK cells.

## 2. Results and discussion

### 2.1 Stochastic loading of traps to achieve variable E : T ratios

**Fig. 2:**
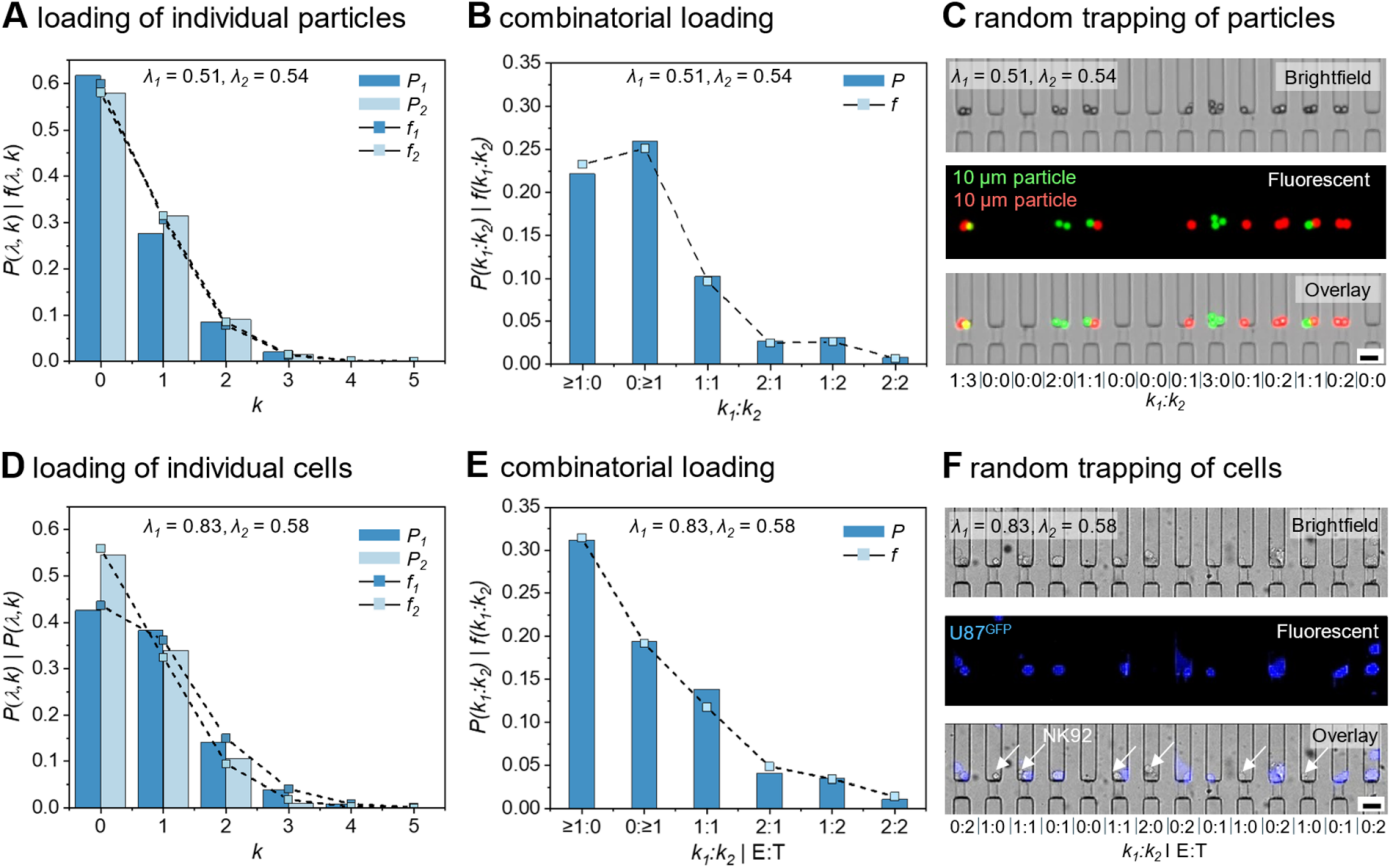
Characterization of the CellTrap device with 1024 traps. (A) The experimental loading frequencies (*f*) of individual particles, *i.e.*, green and red, are compared to the theoretical Poisson distributions (*P*) for *λ*_1_ = 0.51 (green) and *λ*_2_ = 0.54 (red). (B) A combinatorial loading distribution of the two particle types was analyzed using a double Poisson distribution, which is compared with the experimental dual loading frequency. (C) Representative bright-field, fluorescence, and overlay images of red and green particles trapped in a channel at different *k*_1_ : *k*_2_ ratios. (D) The experimental loading frequencies (*f*) and the theoretical Poisson distributions (*P*) are plotted for the U87^GFP^ and NK92^IL2^ cells seeded in the CellTrap device with *λ*_1_ = 0.83 (U87^GFP^) and *λ*_2_ = 0.58 (NK92^IL2^). (E) The experimental dual loading frequency and theoretical double Poisson distributions result in varying E : T or *k*_1_ : *k*_2_ ratios. (F) Representative bright-field, fluorescence, and overlay images of cancer (purple) and immune (white arrows) cells trapped inside a channel at different E : T ratios. (scale bars: 30 µm).

The trapping of particles or cells in the CellTrap platform follows a Poisson distribution due to the stochastic nature of their flow. First, we characterized the CellTrap device using 10 µm fluorescent microparticles and subsequently validated the device with cells (Fig. 2). We evaluated the device's performance numerically and experimentally under various operating conditions, including pump *versus* pipette loading, mixed *versus* non-mixed samples, and different filter numbers (Fig. S1). No significant deviation from the analytical Poisson distribution was observed under any condition (Fig. S2). Therefore, pipette-based loading with pre-mixed samples was selected for subsequent experiments due to its operational simplicity and reduced risk of cell damage.

Importantly, while the symmetric bifurcation network ensures uniform flow distribution during initial loading, the sequential occupancy of traps alters local hydraulic resistance. As demonstrated by our numerical modeling (Fig. S1), the hydrostatic pressure-driven flow naturally diverts toward unoccupied traps. This fluidic diversion ensures that once cells are paired, their local microenvironment shifts to a near-static, ultra-low shear state, protecting the cells from flow-induced mechanical stress and ensuring comparable physiological conditions across all occupied traps throughout the 14-hour assay.

Sample loading can be adjusted by varying the initial sample concentration and seeding duration, which determine the distribution of trapped particles or cells across the 1024 traps. After the sample loading, an average number of particles per trap (*λ* = *N*_p_/*N*_t_) is measured for each experiment, where *N*_p_ is the total number of trapped particles in the device and *N*_t_ (=1024) is the total number of traps. This *λ* value is used to evaluate the analytical Poisson distribution *P*(*λ*, *k*) to compare with the experimental frequency *f* (*λ*, *k*) of loading events for a given trap (*k*). The theoretical probability of *k* for a known *λ* is given by *P*(*λ*, *k*) = (*λ*^*k*^ e^−*λ*^/*k*!), where *k* = 0, 1, 2, … is the number of particles in one trap. The experimental frequency of *k* is calculated as *f*(*λ*, *k*) = *n*_t_(*k*)/*N*_t_, where *n*_t_ is the number of traps for a given *k* = 0, 1, 2, ….

We seeded a mixture of two particles, *i.e.*, green (1) and red (2), into the CellTrap device and measured their experimental loading frequencies, *f*_1_ and *f*_2_, respectively, across the 1024 traps, which aligned well with the theoretical probabilities (*P*_1_ and *P*_2_) for both particles, respectively (Fig. 2A–C). Here, a *λ*_1_ = 0.51 resulted in more than half of the traps empty of green particles (*i.e.*, *n*_t,1_(0) = 632, *f*(0) = 62%), whereas 28% of the traps had a single green particle loaded (*i.e.*, *n*_t,1_(1) = 283), matching the theoretical predictions (Fig. 2A). The remaining 10% of the traps were occupied by two or more green particles per trap. A similar trend is observed for the red particles with *λ*_2_ = 0.54. By varying *λ*, the theoretical probabilities for different values of *k* can be evaluated (Fig. S3). When the experimentally observed frequencies of *k* agree with these theoretical probabilities, the experimental distribution can be predicted and tuned both for single and multiple loadings, according to the theoretical trend.

A combinatorial loading distribution of the two particle types was analyzed using a double Poisson distribution, defined as: *P*(*k*_1_ : *k*_2_) = *P*(*k*_1_) × *P*(*k*_2_) for corresponding means *λ*_1_ and *λ*_2_ (Fig. 2B). For comparison, the experimental dual loading frequency was determined as: *f*(*k*_1_ : *k*_2_) = *f*(*k*_1_) × *f*(*k*_2_). Approximately 10% of the traps contained exactly one particle of each type, *i.e.*, *f*(1 : 1) ≈ 0.1, corresponding to *n*_t_(1 : 1) = 105. Traps with *k*_1_ : *k*_2_ = 1 : 2 and 2 : 1 each accounted for approximately 3% of the total traps, *i.e.*, *n*_t_(1 : 2) = 32, *n*_t_(2 : 1) = 30. A small fraction of traps (<2%) contained multiplets with *k*_1_ : *k*_2_ = (≥2 : 2). A majority of the traps ≈38% were empty (*i.e.*,*k*_1_ : *k*_2_ = 0 : 0, *n*_t_(0 : 0) = 391). The remaining traps with *k*_1_ : *k*_2_ = 0 : ≥1 (*n*_t_ = 241) and ≥1 : 0 (*n*_t_ = 202) were accounted as control groups. The experimental value of *f*(1 : 1) = 10% matched the theoretical prediction of 9.2% double (1 : 1) occupancies using *λ*_1,2_ = 0.5 by the Poisson distribution (Fig. S3). Experimental images of the CellTrap devices confirmed these variable *k*_1_ : *k*_2_ ratios of the loaded particles (Fig. 2C). Diverse *k*_1_ : *k*_2_ ratios were achieved within the same device, enabling a mixed population of empty (0 : 0), control (0 : ≥1 or ≥1 : 0), multiplet (≥1 : ≥1, except 1 : 1), and singleton (1 : 1) traps, with their proportions adjustable by varying *λ*.

We next extended this analysis to living cells. U87^GFP^ (*λ*_1_ = 0.83) and NK92^IL2^ (*λ*_2_ = 0.58) cells exhibited similar stochastic loading behavior (Fig. 2D). Approximately 13.8% of the traps contained exactly one cell of each type, *i.e.*, *f*(1 : 1) ≈ 0.138, corresponding to *n*_t_(1 : 1) = 142, closely matching the theoretical prediction of 11.8% for double (1 : 1) occupancies based on *λ*_1_ = 0.83 and *λ*_2_ = 0.58 (Fig. 2E). Traps with symmetric pairings, *k*_1_ : *k*_2_ = 1 : 2 and 2 : 1, each represented about 4% of the total, *i.e.*, *n*_t_(1 : 2) = 36, *n*_t_(2 : 1) = 42, whereas fewer than 2% contained multiplets with *k*_1_ : *k*_2_ = (≥2 : ≥2). About 22% of the traps were empty, *i.e.*, *k*_1_ : *k*_2_ = 0 : 0, *n*_t_(0 : 0) = 230. The remaining traps, classified as controls, contained cells of only one type, *i.e.*, *k*_1_ : *k*_2_ = 0 : ≥1 (*n*_t_ = 211) and ≥1 : 0 (*n*_t_ = 146). Random trapping of fluorescently labeled U87^GFP^ cells with the NK92^IL2^ cells visually confirmed these distribution patterns (Fig. 2F). A single experiment enables a wide variety of E : T ratios. The number of empty, singletons, and multiplets for particles (*λ*_1_ = 0.51 and *λ*_2_ = 0.58) and for cells (*λ*_1_ = 0.83 and *λ*_2_ = 0.58) is represented in Table S2.

### 2.2 Immune cell–tumor interactions

#### 2.2.1 Functional response of PBMCs against U87^GFP^ cells

**Fig. 3:**
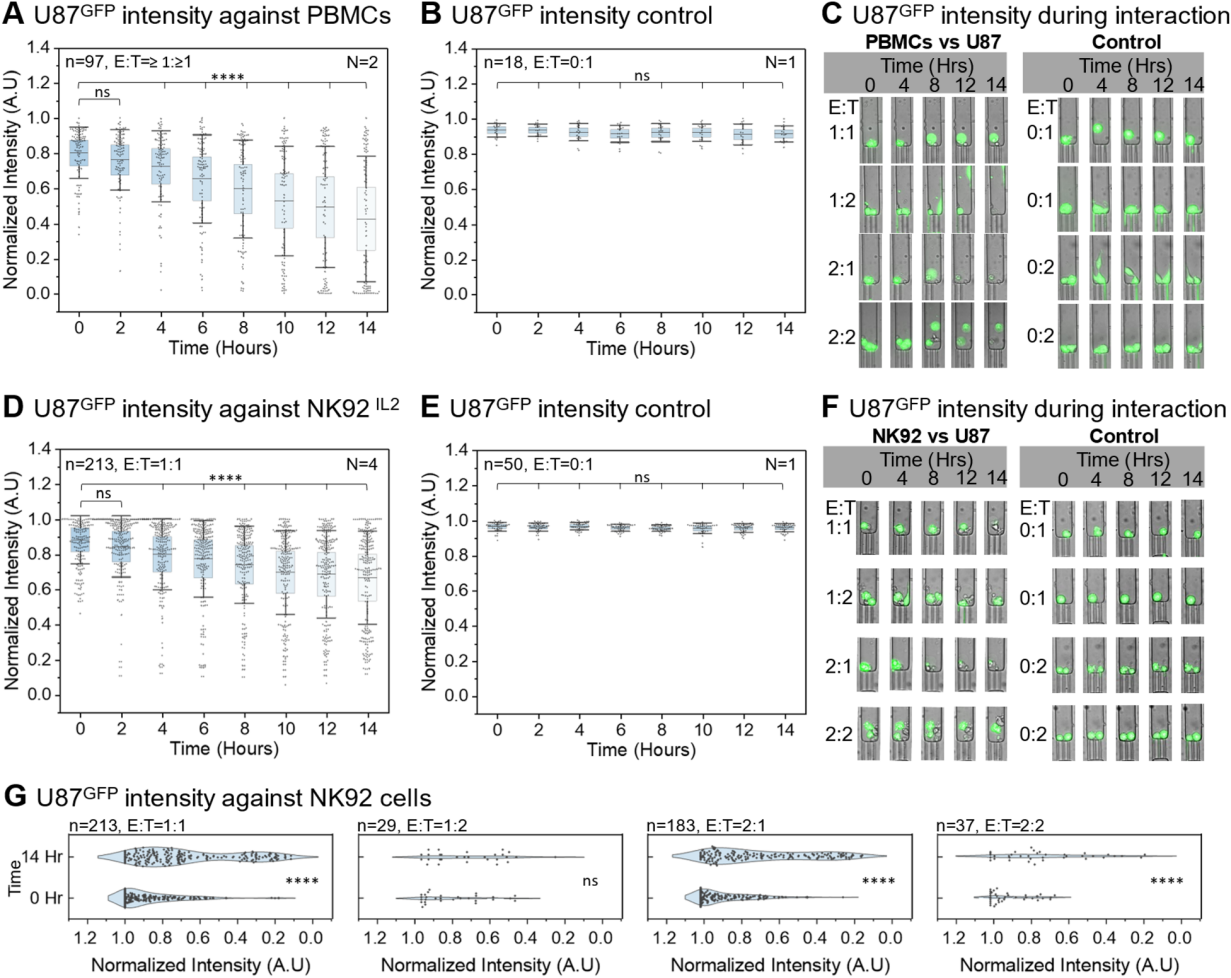
Response of PBMCs and NK92^IL2^ against U87^GFP^ cells. (A) Fluorescence intensity of U87^GFP^ cells decreases significantly after 4 h of co-incubation with PBMCs at E : T = ≥1 : ≥1. This data is curated from two independent CellTrap devices (*N* = 2), where, in total, 97 traps were analyzed (*n* = 97). (B) Inside one of the CellTrap devices used in (A), control traps with only one U87^GFP^ cell per trap, *i.e.*, E : T = 0 : 1, were analyzed, maintaining a stable fluorescence signal over 14 h (*N* = 1, *n* = 18). (C) Representative time-lapse images of U87^GFP^ cells interacting with PBMCs at different E : T ratios, along with the control group containing only U87^GFP^ cells. (D) Fluorescence intensity of U87^GFP^ cells decreases significantly after 4 h of co-incubation with NK92^IL2^ at E : T = 1 : 1. This data is curated from four independent CellTrap devices (*N* = 4), where, in total, 213 traps with E : T = 1 : 1 were analyzed (*n* = 213). (E) Inside one of the CellTrap devices used in (D), control traps with only one U87^GFP^ cell per trap, *i.e.*, E : T = 0 : 1, were analyzed, maintaining a fluorescence signal over 14 h (*N* = 1, *n* = 50). (F) Representative time-lapse images of U87^GFP^ cells interacting with NK92^IL2^ at different E : T ratios, along with the control group containing only U87^GFP^ cells. (G) Fluorescence intensity of U87^GFP^ at 0 h and 14 h of co-incubation with NK92^IL2^ at E : T = 1 : 1, 1 : 2, 2 : 1 and 2 : 2. The intensity drop is significant across all E : T ratios except 1 : 2. This data is curated from the same CellTrap devices used in (D).

To demonstrate the platform's capacity to handle clinically relevant primary samples, PBMCs were co-cultured inside our CellTrap device with U87 glioblastoma cells (U87^GFP^) to evaluate immune–cancer cell interactions (Fig. 3A–C and Movie S1). The response of U87^GFP^ cells, in the presence and absence of PBMCs, was monitored by tracking their fluorescence intensity over a 14-hour period. As the PBMCs interacted with the U87^GFP^ cells, the fluorescence signal gradually weakened and eventually disappeared. This signal loss, corroborated by visible morphological changes in brightfield imaging (*e.g.*, membrane blebbing and structural bloating), confirmed target cell lysis (Fig. 3A). However, this diminishing fluorescence effect was not observed in the control group of U87^GFP^ cells, which were not co-incubated with PBMCs (Fig. 3B). Representative examples of PBMCs interacting with U87^GFP^ cells at different E : T ratios highlight a heterogeneous response (Fig. 3C). For example, at an E : T ratio of 1 : 1, cancer cell membrane integrity was compromised after 8 h, indicating that the cell was moving towards an apoptotic state, even though the green fluorescence signal had not yet completely diminished. For an E : T ratio of 1 : 2, one cancer cell was neutralized by the immune cell after 12 h, whereas the second cancer cell migrated away from the immune cell (note: to maintain quantitative rigor, traps exhibiting such escape events were systematically excluded from bulk E : T cohort analyses). For an E : T ratio of 2 : 1, in the presence of two immune cells, the membrane of the single cancer cell was compromised after 8 h, as the cell initially transformed into a bloated phenotype before completely losing the fluorescence signal. For an E : T ratio of 2 : 2, both cancer cells were neutralized by 12 h, whereas a clear lag in their degree of apoptosis was apparent from the varying fluorescence signal. As an on-chip control, cancer cells maintained in the absence of PBMCs remained morphologically intact and retained stable fluorescence intensity throughout the observation period (Fig. 3C).

While resting PBMCs are largely non-reactive, the robust cytotoxicity observed against U87^GFP^ targets here is attributed to the 24-hour pre-stimulation with IL-2, which is known to activate the NK cell fraction and induce Lymphokine-Activated Killer (LAK) activity. However, to perform rigorous technical characterization of the platform across multiple variables (*e.g.*, dose-responses and temporal calcium signaling), it was necessary to decouple the inherent phenotypic heterogeneity of this bulk population from device-induced technical variability. Therefore, we subsequently transitioned to the standardized NK92^IL2^ cell line to enable precise, reproducible E : T ratio mapping for the remainder of the study (Section 2.2.2).

#### 2.2.2 Functional response of NK92^IL2^ cells against U87^GFP^ cells

Initially, primary human PBMCs were used as effector cells against U87 targets to demonstrate the platform's physiological relevance and its ability to handle heterogeneous, clinically relevant samples (Fig. 3A–C). However, to perform rigorous technical characterization of the platform across multiple variables (*e.g.*, precise E : T dose-responses, cross-target comparisons, and temporal calcium signaling), it was necessary to decouple donor-derived biological heterogeneity from device-induced technical variability. Therefore, we subsequently employed the NK92^IL2^ cell line as a standardized, homogeneous effector population. This phased approach enabled highly reproducible and mechanistically interpretable validation of the platform's analytical capabilities (Fig. 3D–G and Movie S2).

Prior to quantifying cytotoxicity, we performed baseline viability and proliferation studies to ensure the biocompatibility of the CellTrap microenvironment. In control traps containing strictly immune cells or strictly cancer cells (*i.e.*, no target engagement), cells proliferated normally and maintained a high baseline viability (>93%) over the 14-hour period (Fig. S4). This confirmed that the microfluidic confinement itself does not induce spontaneous cell death.

The interaction between NK92^IL2^ and U87^GFP^ cells at a 1 : 1 E : T ratio showed a statistically significant reduction in the fluorescence intensity of the cancer cells starting after 4 hours (Fig. 3D). Moreover, a significant downward shift in the U87^GFP^ cell population, accompanied by a decrease in average fluorescence intensity from 0.88 a.u. to 0.66 a.u., was evident after 14 hours, indicating an average of nearly 22% cancer cell death. Comparison with the target-only control group demonstrated the immune cells exert a highly specific, contact-dependent killing effect on the cancer cells (Fig. 3E). The effect of varying E : T ratios (NK92^IL2^*vs.* U87^GFP^) highlights a dose-dependent kinetic response similar to that observed with PBMCs (Fig. 3F). At an E : T ratio of 1 : 1, loss of cancer cell membrane integrity was typically observed after 12 h of co-incubation with a single immune cell. For an E : T ratio of 1 : 2, one of the two cancer cells was eliminated by the single NK92^IL2^ cell after 8 h, whereas the second cell remained viable throughout the experiment until 14 h. Conversely, for an E : T ratio of 2 : 1, the increased effector presence accelerated target lysis, with cancer cell death occurring earlier, at 4 h. At a 2 : 2 ratio, cooperative dynamics were observed: one cancer cell was neutralized after 8 h, while the other persisted until 14 h. As an on-chip control, U87^GFP^ cells maintained in the absence of NK92^IL2^ cells remained morphologically intact and retained stable fluorescence intensity throughout the 14-hour observation period. A significant population shift in U87^GFP^ cells was observed after 14 hours of incubation with NK92^IL2^ cells across the different E : T ratios, with the exception of the 1 : 2 cohort (Fig. 3G). In particular, E : T ratios of 1 : 1 and 2 : 1 were associated with a marked reduction in U87^GFP^ fluorescence intensity, indicative of substantial target cell killing. At the E : T ratio of 1 : 2, a discernible shift toward reduced U87^GFP^ intensity was also observed in traps where at least one cancer cell underwent cell death. However, the overall change was not statistically significant when averaging the signal across both target cells within the trap, which could be due to the limited number of stable data points (*n* = 29) remaining after applying our strict exclusion criteria for dividing or escaping cells.

#### 2.2.3 NK92^IL2^-mediated cytotoxic response against K562, LS174T, and U87 cells

##### 2.2.3.1 Short-term calcium responses

**Fig. 4:**
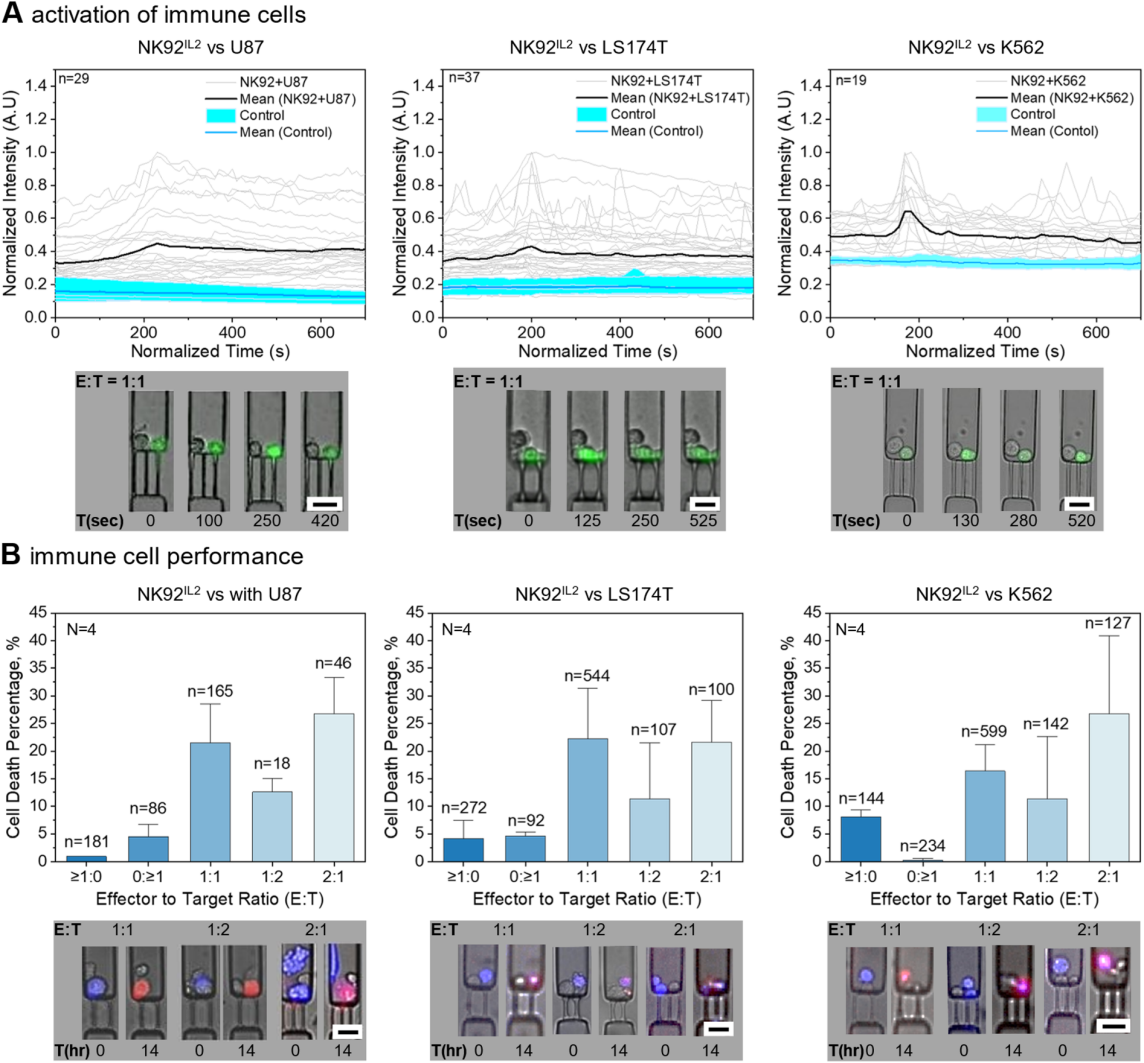
Calcium flux and killing response of immune cells against cancer cells. (A) Calcium flux (normalized intensity) in NK92^IL2^ immune cells varies over time in the presence of various cancer cell lines (U87, LS174T, K562). NK92^IL2^ cells alone show a flat response (control). Each grey line represents a single immune cell tracked. Representative images show NK92^IL2^ cells (green) and cancer cells (U87, LS174T, K562) co-incubated in the CellTrap chip at an E : T ratio of 1 : 1. *n* = number of traps analyzed. (B) The killing response of NK92^IL2^ cells at different E : T ratios (1 : 1, 1 : 2, 2 : 1) against cancer cells (U87, LS174T, K562) is quantified and compared with control groups with E : T ratios of ≥1 : 0 and 0 : ≥1. *N* = number of CellTrap chips analyzed. *n* = number of traps analyzed. Representative images show NK92^IL2^ cells interacting with cancer cells (U87, LS174T, K562 in blue) with varying E : T ratios at 0 and 14 hours. Red color indicates cell death at 14 hours. Scale bars: 25 µm.

One of the earliest activation events in immune cells upon recognizing a target cancer cell is a transient increase in intracellular calcium, which is essential for cytokine secretion and cytotoxic granule-mediated killing of cancer cells.^49–51^ To evaluate this calcium response, NK92^IL2^ cells are loaded with the Fluo-4 calcium imaging reagent. The labeled NK92^IL2^ cells are then incubated either alone or in co-culture with cancer cells at varying E : T ratios, and their interactions are recorded every 10 seconds for 30 minutes using an environmentally controlled fluorescence microscope (Fig. 4A and Movie S3).

When incubated with U87 cells, NK92^IL2^ cells exhibited a gradual rise and fall in calcium flux, with single-cell traces (grey lines) showing transient peaks that were largely absent in NK92^IL2^-only controls (cyan). This slower decline may reflect differences in the ligands presented by U87 cells, the stability of their physical and functional engagement with NK92^IL2^ cells, or the duration of Ca^2+^ signaling engagement. In contrast, incubation with LS174T cells produced a mixture of calcium responses in NK92^IL2^ cells, whereas K562 targets induced a pronounced, sudden spike in calcium flux in immune cells. These differences in calcium peak dynamics likely reflect distinct modes of NK92^IL2^ signaling and activation upon contact with each cancer type. Because K562 cells lack MHC class I, NK92^IL2^ recognition follows the “missing-self” principle. In contrast, U87 and LS174T cells express MHC class I, so NK92^IL2^ activation depends primarily on the balance between activating and inhibitory receptor engagement, with both target types presenting higher levels of activating than inhibitory ligands.^52^ Control traces (cyan) remained flat in all cases, confirming that calcium flux transients in the immune cells were specifically induced by the engagement with the target cells.

Beyond distinct activation profiles across different target cell lines, the single-cell resolution of the CellTrap platform revealed pronounced heterogeneity in effector responses even within the same microenvironment. As illustrated in Fig. S7A, when multiple NK92^IL2^ cells were co-incubated with target cells, individual effectors exhibited distinctly different functional states; for instance, one effector could trigger a robust physiological calcium flux while an adjacent immune cell remained entirely non-responsive over the same observation window. Such localized functional variations underscore the critical value of single-cell monitoring, as these discrete activation dynamics are completely obscured in bulk-averaged assays.

In addition to these heterogeneous physiological fluxes, a stagnant high-intensity response was observed in a few traps, representing immune cells that were compromised or underwent cell death shortly after loading (Fig. S7B). To ensure that traces representing active target engagement were not confounded by these non-specific signals, we characterized the distinct calcium signature of compromised effectors to establish clear exclusion criteria. As illustrated in Fig. S7B, cells undergoing death exhibit a prolonged, stagnant high-intensity calcium influx, likely resulting from the catastrophic loss of membrane integrity and the subsequent disruption of intracellular calcium stores. This “death signature” is sharply distinct from the physiological, transient peaks characteristic of healthy effectors actively engaging with their targets.

##### 2.2.3.2 Extended cytotoxicity response dynamics

The immune response was further evaluated within the CellTrap device by using the NK92^IL2^ immune cells against the three cancer cell lines (Fig. 4B). To ensure unambiguous quantification of target-specific cytotoxicity and to prevent the misattribution of immune cell exhaustion as target cell death, a dual-fluorescence discrimination strategy was employed. Target cells (U87, LS174T, K562) were pre-labeled with a stable fluorescent tracer (CellTracker) that is retained post-lysis. Concurrently, propidium iodide (PI), a bona fide membrane-impermeable death marker, was included in the continuous culture medium. Consequently, target cell death was strictly defined by the spatial colocalization of the blue tracer and the red PI signal (yielding a magenta composite signal, Fig. 4B). Unlabeled NK92^IL2^ cells undergoing spontaneous or activation-induced cell death incorporated PI but lacked the blue tracer, allowing precise, decoupled quantification of both target and effector viability within the same trap.

Additionally, stringent data filtering criteria were applied to preserve E : T ratio integrity. During the near-static incubation, adherent target cells attached to the untreated glass substrate. While LS174T cells remained stably confined, the highly motile U87 cells occasionally utilized this substrate attachment to crawl and squeeze through the trap constrictions. Any trap in which a cell escaped was strictly excluded from the final quantitative analysis. Furthermore, continuous time-lapse imaging allowed the exclusion of cells that underwent division during the observation window. To maximize data yield in future experiments utilizing highly proliferative target lines, researchers can easily integrate standard pre-treatments, such as Mitomycin C or gamma irradiation, prior to hydrostatic loading to achieve complete target cell growth arrest without compromising viability.

With these rigorous parameters established, the killing efficiency of NK92^IL2^ cells was compared across different E : T ratios. To rule out device-induced cytotoxicity, we first verified baseline cell viability and proliferation in the absence of target engagement (Fig. S4). The same-chip and separate-chip controls demonstrated high cell viability (>93%), highlighting that cell death in co-cultured traps results strictly from immune-mediated killing rather than spontaneous stress.

For U87 cells co-incubated with NK92^IL2^ cells at a strict 1 : 1 E : T ratio, we observed an average cancer cell death of ∼21.5%, representing optimal cell–cell interaction and sustained physical engagement. When the E : T ratio was reduced to 1 : 2, the cytotoxicity response decreased by nearly half, to ∼12.6%, indicating that the limited availability of effector cells restricted NK92^IL2^ engagement. Conversely, increasing the E : T ratio to 2 : 1 enhanced the cell death rate to ∼26.7%, suggesting that higher effector density improves the probability of contact and promotes cumulative target lysis.^53^ We observed a cell death of ∼1% for the effector-only (≥1 : 0) and ∼4.4% for the target-only (0 : ≥1) internal control groups, confirming minimal spontaneous apoptosis.

For LS174T cells at a 1 : 1 E : T ratio, cancer cell death peaked at ∼22%, decreasing to ∼11.3% for a 1 : 2 ratio. However, increasing the E : T ratio to 2 : 1 did not significantly enhance the killing response (∼21.4%). Similarly, the K562 cells co-incubated at E : T ratios of 1 : 1, 2 : 1, and 1 : 2 resulted in ∼16.4%, 27%, and 11.3% cell death, respectively.

Notably, the continuous high-frequency imaging required for calcium tracking induces significant photobleaching over prolonged periods. Therefore, early calcium dynamics and 14-hour cytotoxicity were quantified in parallel, independent assays. Interestingly, while the diverse ligand profiles of K562 (missing-self) and U87/LS174T (activating ligand dominance) generated distinct early calcium signatures, their 1 : 1 endpoint killing efficiencies converged within a similar range (∼16–22%).

It is also noteworthy that the observed 1 : 1 killing efficiency against K562 targets (∼16.4%) is lower than some values reported in highly confined droplet-based microfluidic studies. This variance is primarily attributed to our conservative tracking methodology, which strictly excludes dividing target cells to maintain pure E : T ratios, and the open-channel architecture of the CellTrap. Unlike oil-sealed droplets that physically force continuous cell–cell contact and artificially concentrate secreted lytic factors, our platform provides geometric freedom, thereby strictly measuring active, contact-dependent target engagement and limiting artificially amplified bystander killing.

While this compartmentalized design deliberately prevents undefined trap-to-trap crosstalk, which is a reductionist approach necessary to isolate direct contact-dependent killing mechanisms, it inherently restricts the complex, tissue-wide paracrine signaling present in physiological tumor microenvironments. However, because the stochastic loading generates variable, higher-order multi-cellular pairings (*e.g.*, 2 : 1 or 2 : 2 E : T ratios), the CellTrap device allows researchers to observe localized cooperative behaviors and co-activation among small, defined populations of neighboring immune cells that strict 1 : 1 pairing devices cannot capture.

Finally, a primary advantage of the CellTrap architecture over sealed droplet systems is the ability to perform sequential reagent exchange directly on-chip. To demonstrate this capability, we executed a multi-step intracellular Granzyme B assay following 8 hours of live co-culture (Fig. S5). This process required the sequential perfusion of fixation and permeabilization buffers, intermediate washes, and fluorescent antibody labeling. The platform successfully retained the stochastically paired cells throughout these fluidic exchanges. As validated against bulk well-plate controls, NK92 cells treated with an isotype control (IgG) showed no background signal, whereas those stained for Granzyme B exhibited strong intracellular localization. In co-cultured traps, both NK92 effectors and K562 targets maintained their spatial confinement and specific fluorescent signatures throughout the rigorous washing protocols. While further optimization is required to quantify low-abundance cytokine transfer into target cells, this experiment validates the platform's utility for combining long-term live-cell observation with complex, multi-step endpoint phenotypic profiling.

## 3. Conclusions

This work presents a microfluidic cell-trapping device that enables long-term imaging and quantitative analysis of immune–cancer cell interactions at single-cell resolution. By establishing a stable, near-static microenvironment during incubation, minimizing crosstalk between neighboring traps, and intentionally leveraging variable effector-to-target (E : T) ratios driven by Poisson loading, the platform addresses key limitations of conventional cytotoxicity assays that rely on bulk, endpoint measurements. The CellTrap device offers a simple, user-friendly workflow capable of instrument-free operation, enabling rapid cell loading within minutes and immediate observation of cellular interactions. Stable time-lapse imaging was achieved for up to 14 hours while maintaining a high baseline viability (>93%) in control groups, confirming that the measured effects arise from biological processes rather than device-induced stress.

Because individual cancer cells are tracked over time using continuous time-lapse imaging, cell death can be strictly attributed to immune-mediated killing by excluding confounding events such as cell division or escape. The same chip simultaneously provides variable E : T ratios alongside matched on-chip controls, reducing batch effects and enabling internally consistent comparisons. To support high-throughput experiments, a custom MATLAB pipeline was developed to analyze 1024 traps in parallel, allowing scalable quantification of interaction dynamics and cytotoxicity kinetics.

Using NK92^IL2^ cells co-incubated with K562, U87, and LS174T cancer cells, we observed dose-dependent cytotoxicity at higher E : T ratios. Notably, while distinct cancer types triggered divergent early calcium signaling patterns, their killing efficiencies at 1 : 1 E : T ratios converged to approximately 16–22%, suggesting a kinetic plateau for single-effector engagement under these conditions. Importantly, early calcium signaling and long-term fluorescence loss provided complementary readouts, capturing both rapid immune activation and delayed cytotoxic outcomes.

Overall, this platform is particularly valuable in scenarios where continuous monitoring and temporal resolution are essential and where FACS-like endpoint assays are insufficient to capture heterogeneity, timing, and interaction history. Finally, the open-channel trap design enables sequential perfusion and multiple medium exchanges, as demonstrated by our on-chip immunostaining assays, opening the door to experimental designs that are challenging to implement with conventional droplet-based segmentation. Adaptation for patient-derived cells could further establish this device as a versatile platform for personalized immunotherapy screening and mechanistic studies of tumor–immune dynamics.

## 4. Materials and methods

### 4.1 Device design

We have designed a multistage (*S*) bifurcated microchannel with a number (*N*) of cell traps defined as *N* = 4 × 2^*S*^, where the channel, after the last bifurcation, further splits into four sub-channels terminating at the cell traps. For a device with eight bifurcation stages, *i.e.*, *S* = 8, we can calculate the total number of traps as *N* = 4 × 2^8^ = 1024. The design is motivated by uniformly splitting a channel into two at each bifurcation stage to equal the hydraulic flow resistance (*R*_h_) in each sub-branch. The 1024 parallel traps, with a width (*W*_c_) and gap of 30 µm each, were uniformly spaced in a one-dimensional array spanning over 61.44 mm length of the chip, which can be conveniently fitted on a glass slide (25 mm × 75 mm). Each 30 µm wide trap has two or three filter channels with a width (*W*_f_) of 2 µm and a gap of 10 µm. The filter height (*H*_f_) of 4 µm is designed to be smaller than the channel height (*H*_c_) of 40 µm, facilitating cell trapping. The height of these two layers will be controlled during the spin-coating step of the device fabrication.

### 4.2 Device characterization

The device design was experimentally and numerically investigated to evaluate flow distribution and shear stress within the channels under two operational conditions: syringe pump- and pipette-driven flow. To establish the accurate boundary conditions for the numerical simulations, we first evaluated the hydraulic flow resistance (*R*) of the designed CellTrap device experimentally as 1.48 × 10^13^ Pa s m^−3^ (Fig. S8).

Since the flow in the CellTrap device is driven by hydrostatic pressure produced by the liquid column in a 1 mL pipette tip at the device inlet, the inlet pressure was evaluated based on the liquid column height (*h*). For a liquid column height (*h*) of approximately 10 cm, the inlet pressure is calculated as follows: *P* = *ρgh* = 998 Pa, where *ρ* is the density of the aqueous medium, and *g* is the gravitational acceleration. Using a known inlet pressure (*P* = 998 Pa) and measured hydraulic resistance (*R* = 1.48 × 10^13^ Pa s m^−3^) of the CellTrap device, we calculated from the Hagen–Poiseuille relation a total flow rate through the device as *Q* = *P*/*R* = 6.74 × 10^−11^ m^3^ s^−1^ = 4.04 µL min^−1^. Therefore, it takes ∼2 min to load a concentrated cell suspension of ∼10 µL from the pipette tip to the CellTrap device. We further estimated the flow rate through the last bifurcated channel, which opens into four parallel traps, by dividing the total flow rate by 256 channels as follows: 4.04 µL min^−1^ ÷ 256 = 0.0158 µL min^−1^. For the comparison of pump- and pipette-driven flows, the cell loading experiments in Fig. S2 were performed using a total pump-driven flow rate of 5 µL min^−1^, which translates to nearly the same value of 0.0195 µL min^−1^ of flow after the last bifurcation. This served as a reference flow rate for the inlet boundary conditions in the numerical simulations in Fig. S1 and S9.

The cell traps are simulated in COMSOL Multiphysics 6.2, using the Laminar Flow Module, for syringe pump- and pipette-driven flow conditions to evaluate flow distribution (Fig. S1) and shear stress (Fig. S9) when the traps are empty or filled with cells. For the pump-driven flow, the inlet boundary condition was volumetric flow rate, ranging from 0.019 to 0.215 µL min^−1^. After each simulation, the inlet pressure was evaluated for the given flow rate boundary condition and the occupancy situation of the trap. The evaluated inlet pressure values, ranging from ∼100 to ∼6000 Pa, served as inlet boundary conditions to simulate the respective pipette-driven flows.

Simulations revealed that syringe pumps generate consistently higher shear stress within the filters compared to pipette-driven flow, particularly when traps are occupied. As an active source, the pump maintains a fixed flow rate by increasing pressure to overcome the added resistance of a trapped cell, which can compromise cell viability or force cells to squeeze through filters (Fig. S10). In contrast, pipette-driven flow maintains a consistent inlet pressure regardless of trap occupancy. Once a trap is filled, the local flow rate and shear stress are significantly reduced as fluid is naturally diverted to unoccupied, lower-resistance paths. This self-regulating mechanism ensures that trapped cells are protected from excessive mechanical stress and are less prone to escaping the traps after being squeezed. The total flow rate in the device increases only if the liquid column height in the pipette tip is increased.

### 4.3 Device fabrication

A negative photoresist (SU8-3005; Micro Resist Technology, Germany) was spin-coated on a 4-inch silicon wafer using a two-step program to ensure a uniform coating. First, the wafer was spun at 500 rpm for 10 s with an acceleration of 100 rpm s^−1^, followed by 4500 rpm for 30 s with an acceleration of 300 rpm s^−1^, yielding an expected layer thickness of ∼4 µm. The coated wafer was then soft-baked at 95 °C for 2 min. After soft baking, the hotplate was switched off to allow the wafer to cool gradually to room temperature, thereby minimizing thermal stress. A CAD mask of filters, including alignment markers, was prepared in AutoCAD and converted to an instrument-compatible GDS format. An ultraviolet (UV) exposure (365 nm) of the first spin-coated photoresist layer was performed using a maskless laser writer (µMLA, Heidelberg Instruments, Germany) at a dose of 180 mJ cm^−2^. After UV exposure, the wafer was post-baked for 1 min at 65 °C and 1 min at 95 °C, then allowed to cool to room temperature on the hotplate. Next, a second negative photoresist (SU8-3025; Micro Resist Technology, Germany) was applied on top of the first layer using a two-step spin-coating process (500 rpm for 10 s at 100 rpm s^−1^, then 3000 rpm for 30 s at 300 rpm s^−1^ with an expected thickness of the second layer as ∼25 µm). The wafer was soft-baked at 95 °C for 12 min and cooled to room temperature. The CAD mask of the main channels was aligned to the UV-exposed filters in the first layer using the alignment markers. A second UV exposure was carried out at a dose of 100 mJ cm^−2^. The wafer was then post-baked for 1 min at 65 °C and 4 min at 95 °C, followed by gradual cooling to room temperature. Finally, the wafer was developed for 6 min in mr-Dev 600 (Micro Resist Technology, Germany). The heights of the filters and the main channels were measured as ∼4 µm and ∼35 µm, respectively, using a profilometer (Dektak XTR, Bruker Corporation, Billerica, MA, USA). For soft lithography, polydimethylsiloxane (PDMS) was prepared by mixing the elastomer and curing agent at a 10 : 1 ratio. The mixture was poured onto the SU-8 master mold, degassed under vacuum, and cured at 65 °C for 2 h. After curing, the PDMS layer was peeled from the mold, and inlet and outlet holes were created using a 1.2 mm biopsy punch. The PDMS device was then plasma-bonded to a glass slide and placed in an oven at 65 °C for 20 min to strengthen the bond.

### 4.4 Device operation

The device was designed to enable instrument-free and user-friendly operation. The CellTrap device operates solely *via* a hydrostatic pressure gradient and can therefore be driven using a standard 1 mL pipette tip. Prior to use, the chip was decontaminated with ethanol, thoroughly rinsed with aqueous media, and degassed to remove trapped air bubbles from the microchannels.

For continuous analysis of cell viability during long-term live-cell imaging, cells were seeded into the CellTrap device in RPMI 1640 culture medium supplemented with PI. PI (1.0 mg per mL stock solution) was diluted 1 : 40 000 in RPMI 1640. Concentrated cell suspensions were prepared by resuspending each cell type in 50 µL of medium at a density of 100 000 cells per mL. The PI-containing RPMI medium was first loaded into the pipette tip, after which 20 µL of the cell suspension (≈2000 cells) was aspirated into the same tip by rotating the pipette dial anticlockwise. In this configuration, the PI-containing medium acts as a reservoir above the cell sample, preventing dilution and ensuring a concentrated plug of cells reaches the traps. This setup minimizes the required cell volume and enables rapid and efficient seeding supported by the liquid column height in the 1 mL tip.

Once microscopy confirmed that cells were successfully captured in the traps, the 1 mL loading pipette tip was removed to halt the rapid seeding flow. It was replaced with micro-reservoirs consisting of shortened pipette tips (∼1 cm in height) containing ∼20 µL of PI-supplemented RPMI medium at both the inlet and outlet. To ensure sterile experimental conditions and prevent evaporation-induced flow-rate fluctuations, these micro-reservoirs were immediately sealed with tape. This sealing technique establishes a stable, near-static flow equilibrium, providing a consistent, ultra-low shear environment across all parallel traps and experiments. The sealed chip was then mounted on a Leica LAS X fluorescence microscope equipped with a humidified, CO_2_-regulated environmental chamber for sterile time-lapse imaging with adaptive focusing applied every hour.

During the 14-hour incubation period, the liquid column heights in the inlet and outlet micro-reservoirs were carefully balanced to maintain this near-static fluidic state. This minimized flow ensured that locally secreted cytokines and degranulated lytic molecules were retained within the immediate vicinity of the interacting cells, rather than being flushed away. This configuration allowed for the observation of both contact-dependent and localized paracrine cytotoxic mechanisms. Furthermore, the physical separation between parallel channels and the associated diffusion length scales ensured that these locally accumulated molecules did not cause bystander crosstalk in adjacent microchannels.

### 4.5 Cell isolation and culture

PBMCs were isolated from freshly collected heparinized human blood using density gradient centrifugation with Ficoll–Paque (*ρ* = 1.077 g mL^−1^, Cytiva). Whole blood was diluted 1 : 1 with phosphate-buffered saline (PBS; without Ca^2+^ and Mg^2+^) and gently layered over Ficoll in 50 mL conical tubes. Samples were centrifuged at 500×*g* for 30 minutes at room temperature, with the brake disengaged, to allow for optimal gradient separation. The mononuclear cell layer at the plasma–Ficoll interface was carefully aspirated and transferred to a new tube. Cells were washed twice with PBS supplemented with 2% fetal bovine serum (FBS) by centrifugation at 300×*g* for 10 minutes. Purified PBMCs were stimulated with IL-2 (100 IU mL^−1^) and used after 24 hours.

The human cell lines NK92^IL2^, U87, K562, and LS174T were obtained from the American Type Culture Collection (ATCC, Manassas, VA, USA).

NK92^IL2^ and K562 cells were cultured in RPMI 1640 medium (Gibco™, Thermo Fisher Scientific, Waltham, MA, USA) supplemented with 10% fetal calf serum (FCS), 1% penicillin–streptomycin (Pen/Strep, Thermofisher), and 1% glutamine (Thermofisher). U87 and LS174T cells were cultured in high-glucose Dulbecco's Modified Eagle Medium (DMEM, GlutaMAX™ Supplement; Gibco™, Thermo Fisher Scientific, Waltham, MA, USA) supplemented with 10% (v/v) heat-inactivated fetal bovine serum (FBS; Sigma-Aldrich, Merck KGaA, Darmstadt, Germany) and 1% (v/v) penicillin–streptomycin (10 000 IU per mL penicillin and 10 mg per mL streptomycin; Sigma-Aldrich). All cells were maintained at 37 °C in a humidified atmosphere containing 5% CO_2_.

Adherent cell lines (LS174T and U87) were maintained in complete DMEM, while suspension cell lines (K562 and NK92^IL2^) were cultured in complete RPMI medium. NK92^IL2^ were stimulated with Human IL-2 Recombinant Protein (100 IU mL^−1^, PeproTech®, Thermo Fisher) 24 hours before interaction analysis. Adherent cells were passaged by washing once with PBS (Gibco™), followed by detachment using trypsin (Sigma-Aldrich) and incubation for 3 to 5 minutes at 37 °C. After detachment, enzymes were neutralization with fresh complete medium. Suspension cells were collected directly from culture by transferring the cell suspension to centrifuge tubes. For both adherent and suspension cultures, cells were pelleted by centrifugation using centrifuge (Hettich® ROTINA 420R, Andreas Hettich GmbH & Co. KG, Tuttlingen, Germany) at 500×*g*, the supernatant was removed, and cells were resuspended in the appropriate pre-warmed complete medium for downstream experiments.

### 4.6 Calcium assay

For calcium detection, NK92^IL2^ cells were counted and approximately 200 000 cells were pelleted. Fluo-4 AM staining was performed according to the calcium imaging kit protocol (Cat. No. F10489, Thermo Fisher Scientific) by preparing a 1 mL working solution of Fluo-4 AM and adding it to the NK92^IL2^ cell pellet. The cells were incubated at 37 °C for 30 minutes, followed by an additional 30 minutes at room temperature. After staining, the cells were washed three times with PBS (without Ca^2+^ and Mg^2+^) by centrifugation at 500×*g*. The stained NK92^IL2^ cells were then split into two equal fractions: one was mixed with cancer cells (U87, LS174T, or K562), while the other served as a control with NK92^IL2^ cells only. Samples were loaded into the CellTrap microfluidic chip following the device operation protocol described above. Time-lapse imaging was carried out on an environmentally controlled microscope (Leica LAS X Thunder) using a 10× objective, acquiring brightfield and fluorescence images every ∼10 s for 30 minutes, with control and experimental chips imaged in parallel. Image sequences were analyzed in ImageJ (https://imagej.net/ij/), and calcium flux was quantified from changes in Fluo-4 fluorescence intensity.

### 4.7 On-chip granzyme B assay protocol

For the Granzyme B assay, cells were fixed and permeabilized directly on-chip using a fixation/permeabilization buffer (Cat. No. 88-8824-00, Thermo Fisher Scientific). Briefly, cells were incubated with the fixation buffer for 30 minutes, washed *via* hydrostatic perfusion, and then incubated with the permeabilization buffer for an additional 30 minutes. After washing, anti-Granzyme B antibody-APC (clone REA226, Cat. No. 130-120-703, Miltenyi Biotec, Bergisch Gladbach, Germany) or the corresponding isotype control (IgG2a) mouse antibody-APC (Cat. No. B61429, Beckman Coulter, Krefeld, Germany) was added to the interaction and control samples, respectively, and incubated for 1 hour. Following a final extensive wash to remove unbound antibodies, images were acquired using a fluorescence microscope. The successful retention of cells throughout this multi-step fluidic protocol validates the platform's suitability for complex endpoint phenotypic profiling.

### 4.8 Post-processing

To ensure unbiased analysis across the entire device and validate macro-scale trapping efficiency, high-resolution stitched panoramic images of all 1024 traps were captured and manually analyzed at *t* = 0 and *t* = 14 hours (Fig. S6). The number of cancer or immune cells in a live or dead state was counted at a given time step and processed in Excel. For experiments involving fluorescently labeled U87^GFP^ cells (Fig. 3), stitched images of the 1024 traps, obtained every two hours, were post-processed in MATLAB. Brightfield and fluorescence channels from the stitched images were straightened and then cropped. The stitched images were split into 1024 images corresponding to each trap within the CellTrap device. The number of cancer cells in each split image or trap was counted, and the change in fluorescence intensity over time was simultaneously analyzed. For each trap, a representative split image was saved, and intensity traces were retained only for traps containing both immune and cancer cells for subsequent analysis. The number of immune cells in the corresponding traps was manually tallied from the brightfield images to confirm the E : T ratio.

To maintain quantitative rigor and ensure the accuracy of the Effector-to-Target (E : T) ratio analysis over the 14-hour incubation period, continuous time-lapse imaging was utilized to monitor cell division and motility. Any trap in which a cancer cell or immune cell was observed to proliferate was explicitly excluded from the fixed E : T ratio cohorts (*e.g.*, 1 : 1, 1 : 2, 2 : 1). This strict visual filtering ensured that the cytotoxic dynamics reported correspond precisely to the stated initial and final E : T ratios.

While Poisson statistics predicted >100 target-only control traps per array, the final analyzable dataset (*e.g.*, *n* = 18, *n* = 50) was inherently lower due to the application of these strict exclusion criteria. Similarly, any trap in which a cell (particularly the motile U87 line) migrated out of the trap during the 14-hour window was excluded to maintain E : T ratio integrity. Furthermore, brightfield imaging confirmed that the 2 µm filter constrictions successfully retained apoptotic debris within the traps, verifying that the quantified loss of GFP fluorescence was due to biological cell lysis rather than physical flushing of the target cells.

### 4.9 Statistical analysis

All statistical analyses in Fig. 3 were performed using GraphPad Prism version 10 (GraphPad Software, San Diego, CA, USA). Data are presented as mean ± standard deviation (SD) from one to four independent experiments. Differences between groups were assessed using one-way analysis of variance (ANOVA) followed by Tukey (normal distribution) or Kruskal–Wallis and Dunn's multiple comparisons test (nonparametric test). A *p*-value of <0.05 was considered statistically significant. In the figures, significance levels are indicated as shown *p < 0.0332, **p < 0.0021, ***p < 0.0002, and ****p < 0.0001.

## Ethical statement

All experiments were conducted in accordance with protocol 620/21 S-KK (12 July 2021) and were approved by the Ethics Committee of the Technical University of Munich. Informed consents were obtained from healthy human participants of this study.

## Conflicts of interest

There are no conflicts to declare.

## Supplementary Material

RA-016-D6RA02345B-s001

RA-016-D6RA02345B-s002

RA-016-D6RA02345B-s003

RA-016-D6RA02345B-s004

## Data Availability

The data supporting this article have been included as part of the supplementary information (SI), which includes Movies S1–S3 and a PDF file containing captions for Movies S1–S3, Fig. S1–S10, and Tables S1–S2. Supplementary information is available. See DOI: https://doi.org/10.1039/d6ra02345b.
