# Supplementary material for: CellTrap: an instrument-free microfluidic platform for cell–cell interactions at stochastically generated effector-to-target ratios
Source: RSC Adv. 2026 Apr 10;16(21):19024–37. doi: 10.1039/d6ra02345b (PMC13068002; doi:10.1039/d6ra02345b)
Supplement: RA-016-D6RA02345B-s001 [file RA-016-D6RA02345B-s001.pdf]

Supplementary Information (SI) for RSC Advances.
This journal is © The Royal Society of Chemistry 2026

Supporting information

CellTrap: An Instrument-Free Microfluidic Platform for Cell-Cell Interactions at Stochastically Generated Effector-to-Target Ratios

Muhammad Zia Ullah Khan[1], Morteza Hasanzadeh Kafshgari[2], Ali Bashiri Dezfouli[4], Oliver Hayden[2], Gabriele Multhoff[3], Ghulam Destgeer[1*]

[1]Control and Manipulation of Microscale Living Objects, Center for Translational Cancer Research (TranslaTUM), Munich Institute of Biomedical Engineering (MIBE), Munich Institute of Integrated Materials, Energy and Process Engineering (MEP), Department of Electrical Engineering, School of Computation, Information and Technology (CIT), Technical University of Munich (TUM), Einsteinstraße 25, Munich 81675, Germany.

[2]Heinz-Nixdorf-Chair of Biomedical Electronics, TranslaTUM, MIBE, School of Computation, Information and Technology, Technical University of Munich, Einsteinstraße 25, 81675 Munich, Germany.

[3]Experimental Radiation Oncology and Radiobiology, TranslaTUM, School of Medicine, Technical University of Munich, Einsteinstraße 25, 81675 Munich, Germany.

[4]Department of Otolaryngology, Head and Neck Surgery, TUM School of Medicine and Health, Technical University of Munich, 81675 Munich, Germany.

*Corresponding authors: ghulam.destgeer@tum.de

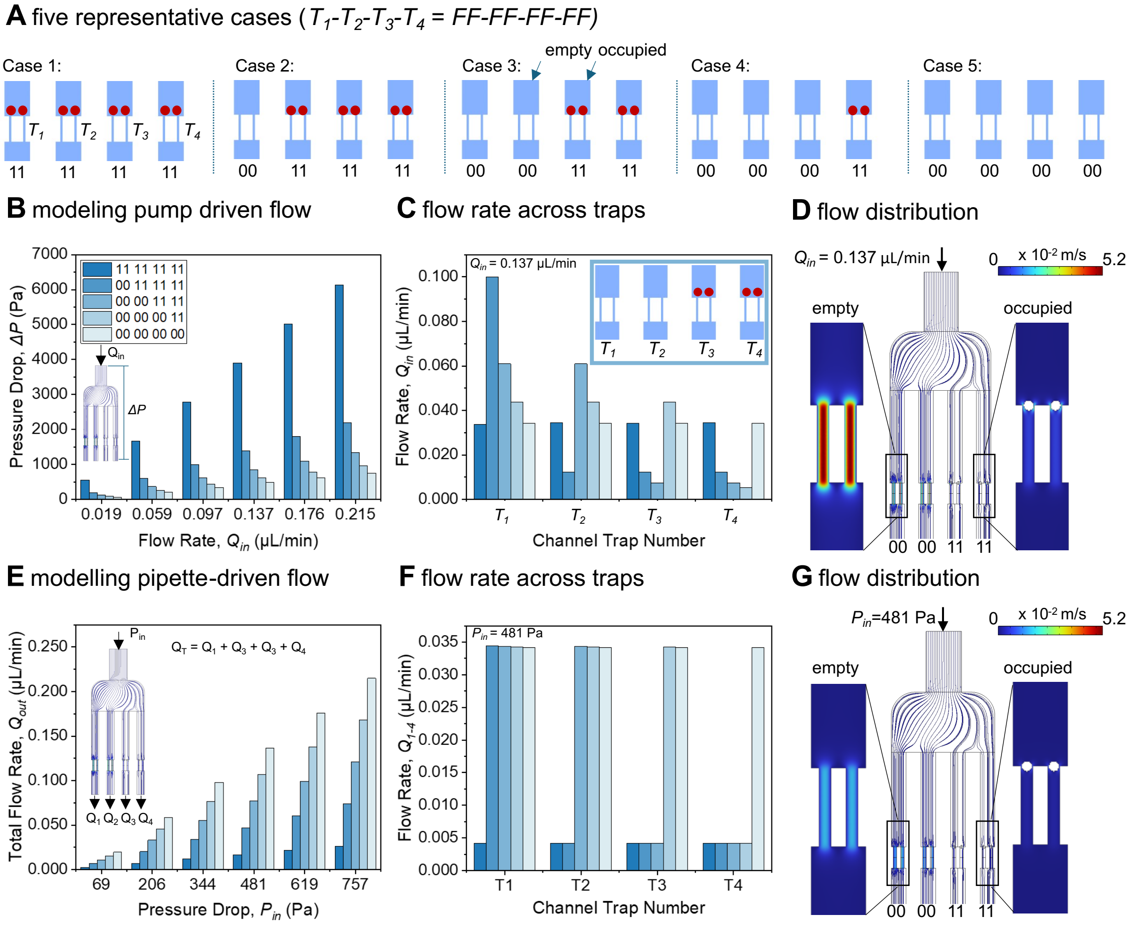

Figure S1. Numerical analysis of flow rate distribution within four parallel daughter channels of a CellTrap device under syringe pump and pipette-driven flow. (A) Five representative cases of traps are defined for this numerical investigation: Case 1: 11-11-11-11, Case 2: 00-11-11-11, Case 3: 00-00-11-11. Case 4: 00-00-00-11, and Case 5: 00-00-00-00. For any given case, each pair of numbers indicates the status of one of the four branches ending in an isolated cell trap (T), such that the case can be represented as T_1-T_2-T_3-T_4. Each trap (T_1-T_4) has two filters (*FF*) that can be in an open (not occupied) or closed (occupied) state; therefore, we can also define the status of the four parallel traps T_1-T_2-T_3-T_4 as *FF-FF-FF-FF*, where F is equal to 0 (open) or 1 (closed). For example, in Case 1: T_1-T_2-T_3-T_4 = 11-11-11-11, all the filters across four traps are occupied by a particle, whereas in Case 5: T_1-T_2-T_3-T_4 = 00-00-00-00, all the filters across four traps are open. (B) For numerical modeling of the flow through the four daughter traps, we first assumed a total flow rate through the device ranging from 5 µl/min to 55 µl/min and then divided the total flow rate by 256 channels after eight bifurcations. This resulted in our inlet boundary condition, in the form of a constant flow rate ranging from 0.019 µl/min to 0.215 µl/min, which mimics an experimental syringe-pump-driven flow, for our simulations. The pressure drop (ΔP) across the filters increased linearly with the inlet flow rate (Q_{in}), as predicted by the Hagen-Poiseuille equation. The highest slope for Case 1: 11-11-11-11 is associated with the highest fluidic resistance of the fully occupied traps. (C) The flow distribution through each daughter trap (T_1-T_4) was further analyzed as a function of trap occupancy by particles. A representative case was evaluated with an inlet boundary condition of Q_{in} = 0.137 µL/min. For Case 1 (T_1-T_2-T_3-T_4 = 11-11-11-11) and Case 5 (T_1-T_2-T_3-T_4 = 00-00-00-00), the flow rates through all four traps remain identical because the hydrodynamic resistance is balanced across the branches. In contrast, for Case 3 (T_1-T_2-T_3-T_4 = 00-00-11-11), the flow rate through traps T_1 and T_2 is higher due to lower flow resistance and the absence of particles. Consequently, branches T_3 and T_4 exhibit substantially reduced flow due to higher flow resistance and the presence of trapped particles. This indicates that, while the syringe pump supplies a constant total flow rate of 0.137 µL/min, the flow is redistributed among the individual traps according to their occupancy, such that the sum of the flow rates through all four traps remains conserved. (D) A streamline and contour representation for Case 3 (T_1-T_2-T_3-T_4 = 00-00-11-11) depicts high velocity (v_{avg} = 29.07 mm/s) for T_1 and T_2 channels and low velocity (v_{avg} = 3.56 mm/s) for T_3 and T_4 channels. A shift in streamlines towards T_1 and T_2 channels can be clearly seen right after the flow enters from the main channel. This highlights that once a trap is occupied, it results in a diversion of flow towards open or non-occupied traps. (E) For the numerical modeling of the pipette-operated flow through the four

daughter traps, we first computed the pressure drop ΔP across the channel for a range of total inlet flow rates Q_{in} from 0.019 µL/min to 0.215 µL/min. This pressure drop was then used as the inlet pressure boundary condition P_{in}, ranging from 69 Pa to 894 Pa. For each P_{in}, the corresponding total outlet flow rate Q_{out} (= $Q_1 + Q_2 + Q_3 + Q_4$) was calculated, and in all cases, the overall flow rate increased monotonically with ΔP. (F) The flow distribution through each daughter trap was further analyzed as a function of trap occupancy by particles. As a representative case, we evaluated an inlet pressure boundary condition of P_{in} = 481 Pa, which corresponds to the pressure drop ΔP obtained for Q_{in} = 0.137 µL/min. The total outlet flow rate Q_{out} (= $Q_1 + Q_2 + Q_3 + Q_4$) differs significantly between two extreme cases, i.e., 0.0168 µL/min for Case 1 (T_1-T_2-T_3-T_4 = 11-11-11-11) and 0.137 µL/min for Case 5 (T_1-T_2-T_3-T_4 = 00-00-00-00), because there is no pump enforcing a fixed flow rate. As expected, for Case 3 (T_1-T_2-T_3-T_4 = 00-00-11-11), the flow rate through traps T_1 and T_2 is higher due to the lower hydraulic resistance in the absence of particles, whereas branches T_3 and T_4 exhibit substantially reduced flow owing to the increased resistance from trapped particles. Unlike in the pump-driven model, each trap in the pipette-operated configuration behaves independently, and the flow through each channel is bounded between a minimum (0.0042 µL/min) and a maximum (0.0342 µL/min) value: the minimum flow rate corresponds to both filters in a trap being occupied, and the maximum flow rate corresponds to both filters being empty. (G) A streamline and velocity contour representation for Case 3 (T_1-T_2-T_3-T_4 = 00-00-11-11) illustrates high velocities (v_{avg} = 16.4 mm/s) in channels T_1 and T_2 and low velocities (v_{avg} = 2.08 mm/s) in T_3 and T_4. Similar to panel (D), a clear diversion of streamlines toward T_1 and T_2 is observed immediately after the flow enters from the main channel, indicating that once a trap is occupied, the flow is redirected toward open (unoccupied) traps. However, the v_{avg} = 16.4 mm/s in the pipette-driven flow (G) is significantly smaller than v_{avg} = 29.07 mm/s in the pump-driven flow (D), highlighting a low shear stress flow suitable for cell trapping without significant cell leakage through the traps. Finally, panels (D) and (G) compare device performance under the same pressure-drop conditions, where (D) corresponds to pump operation and (G) to pipette operation. For the same imposed pressure drop, the velocities in channels T_1 and T_2 of the pump-operated device are significantly higher than those in the pipette-operated device.

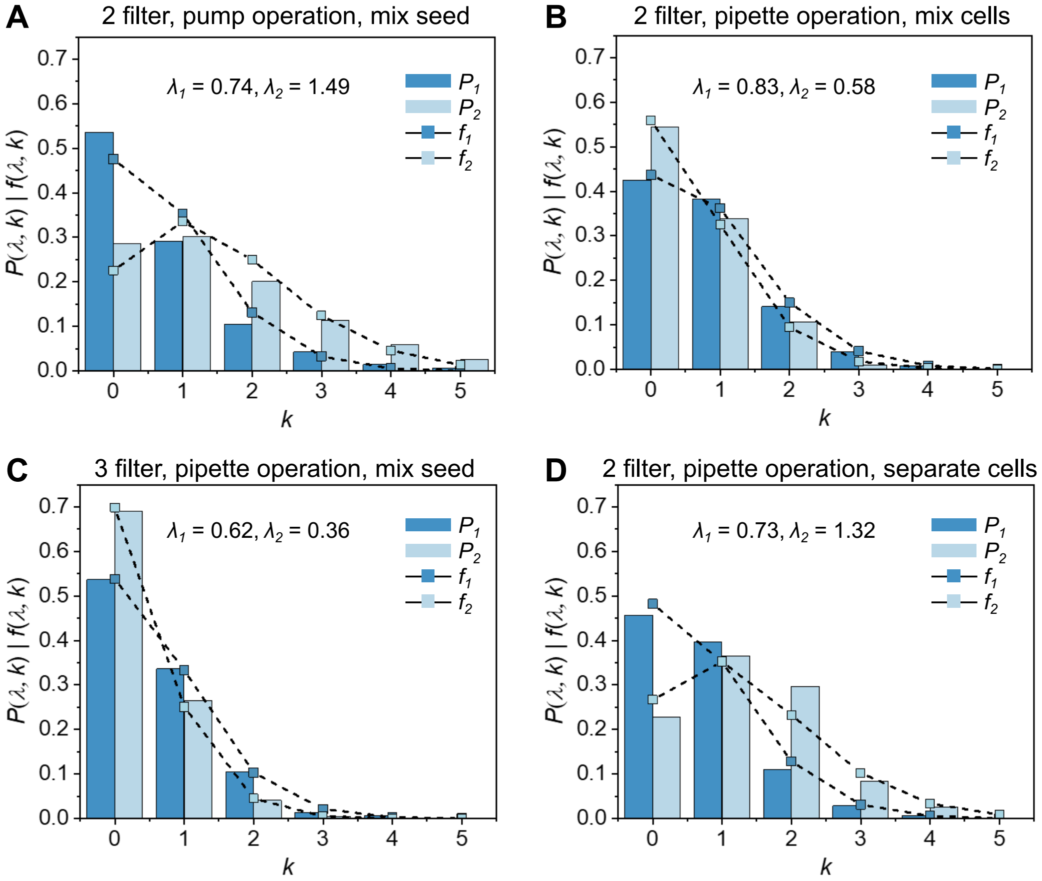

Figure S2. Comparison of experimental and theoretical distribution of immune (NK92^IL2) and cancer (U87^GFP) cells with the CellTrap device for different operating conditions. (A) A premixed sample with λ_1 = 0.74 and λ_2 = 1.49, seeded using a pump, and observed in a trapping device with 2 filter geometries. (B) A premixed sample with λ_1 = 0.83 and λ_2 = 0.58, seeded using a pipette, and observed in a trapping device with 2 filter geometries. (C) A premixed sample with λ_1 = 0.62 and λ_2 = 0.36, seeded using a pipette, and observed in a trapping device with 3 filter geometries. (D) Cancer and immune cells were seeded separately with λ_1 = 0.73 and λ_2 = 1.32, respectively, using a pipette, and observed in a trapping device with 2 filter geometries.

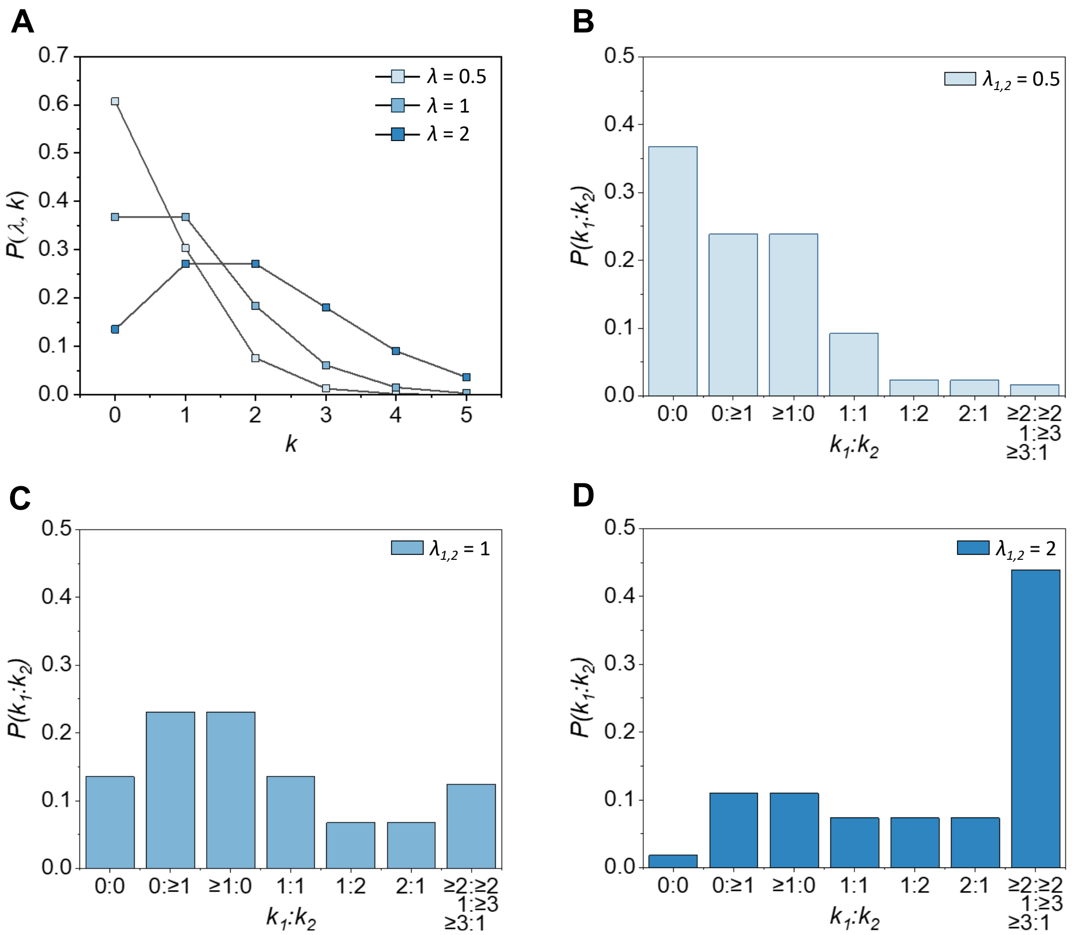

Figure S3. Theoretical Poisson distribution of trapped cells within the CellTrap device at different λ values. (A) As expected, the proportion of multiplets per trap increases with λ as the fraction of empty traps gradually decreases. (B-D) Double loading distribution for $\lambda = 0.5$, 1, and 2.

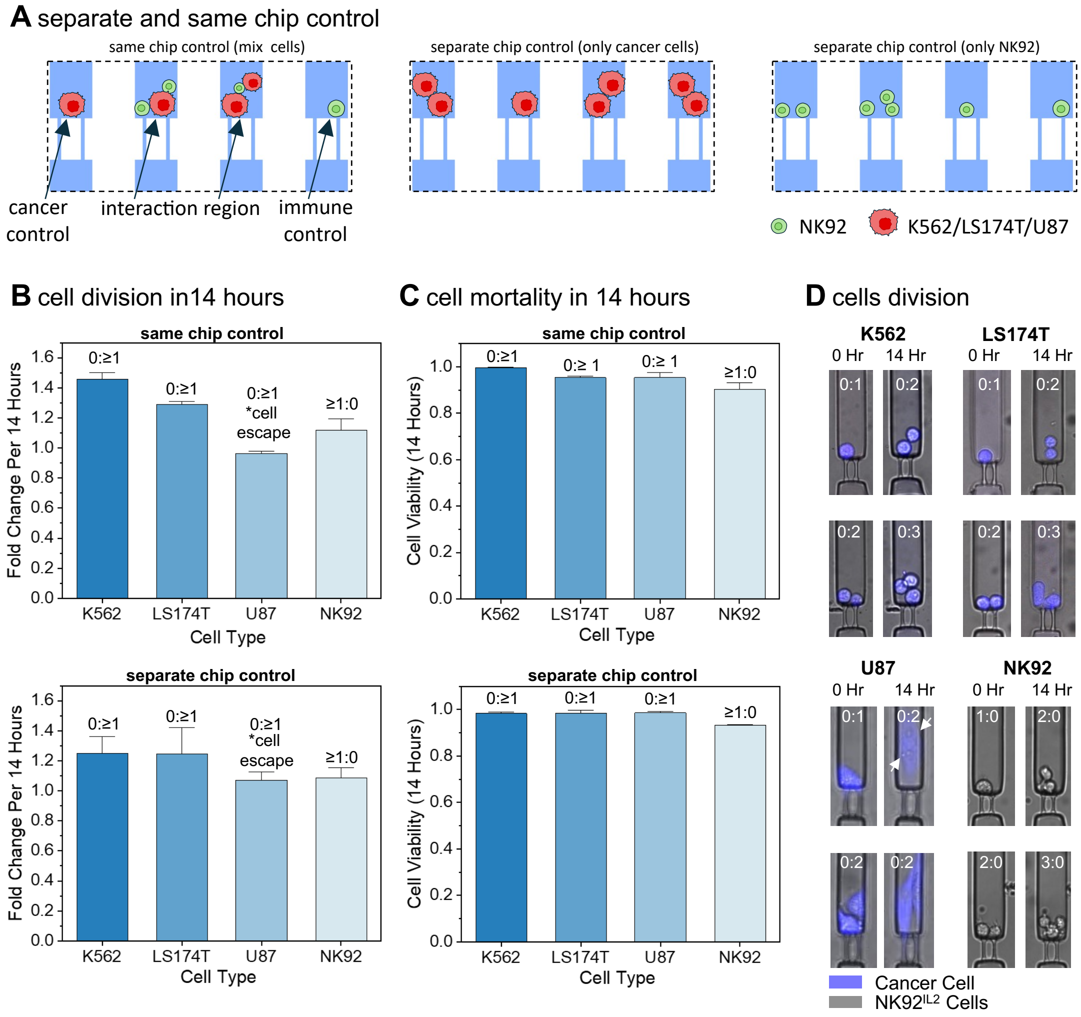

Figure S4. Baseline cell proliferation and viability in separate- and same-chip control conditions. (A) Representative schematic of same-chip and separate-chip controls used to evaluate baseline cellular health. "Same-chip control" refers to traps containing exclusively cancer cells or exclusively immune cells, located alongside interacting co-culture traps within the same device. "Separate-chip control" refers to cancer and immune cells seeded entirely in independent microfluidic chips. (B) Comparison of baseline cell proliferation (fold-change in cell number) after 14 hours of incubation. The lower fold-change recorded for adherent U87 cells is attributed to their motile tendency to escape the trap and field of view post-division. (C) Comparison of baseline cell viability (excluding divided cells) after 14 hours. Crucially, this high viability (>93% for all cell types in both control groups) reflects resting immune and cancer cells cultured in the absence of opposing cell types (i.e., no target engagement). This confirms that the microfluidic confinement itself does not induce spontaneous cell death. (D) Representative time-lapse images illustrating normal cell division over the 14-hour incubation period obtained from the same-chip control.

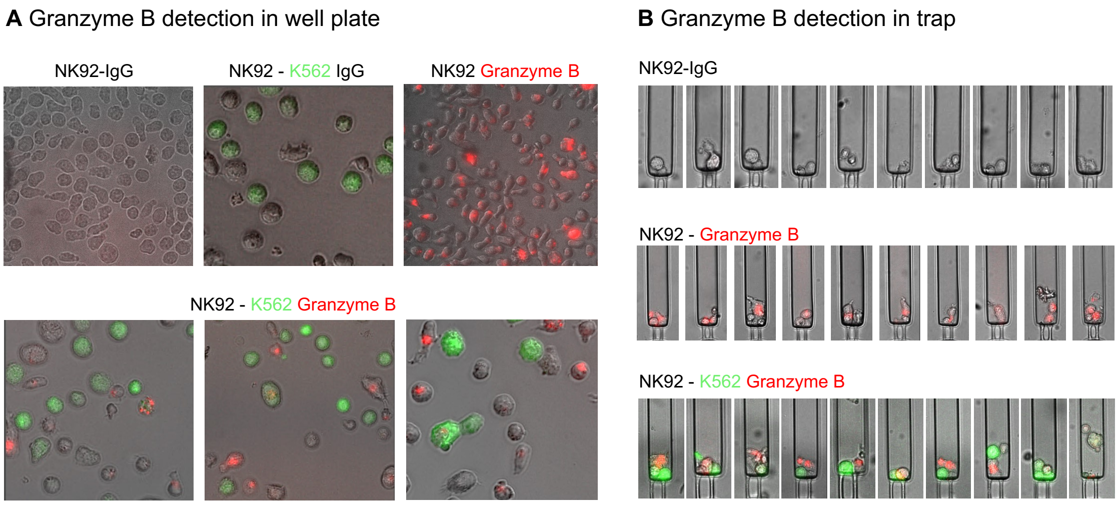

Figure S5. Validation of on-chip medium perfusion via multi-step intracellular immunostaining. (A) Bulk verification in well plates confirming antibody specificity. NK92 cells stained with an isotype control (IgG) show no background signal, whereas those stained with anti-Granzyme B (red) exhibit strong intracellular fluorescence. Co-culture with K562 targets (green) confirms the ability to distinguish the effector phenotype in a mixed population. (B) In-chip detection demonstrating the platform's fluidic retention during sequential reagent exchange. (Top) Negative control (IgG) in traps confirms low background noise in the PDMS environment. (Middle) NK92 cells trapped alone display high intracellular Granzyme B levels (red), serving as the baseline for effector cells. (Bottom) Co-culture of NK92 effectors and K562 targets (green) within single traps, demonstrating successful cell retention and specific staining following fixation, permeabilization, and multiple washing steps.

A 0 Hour (NK92 vs K562)

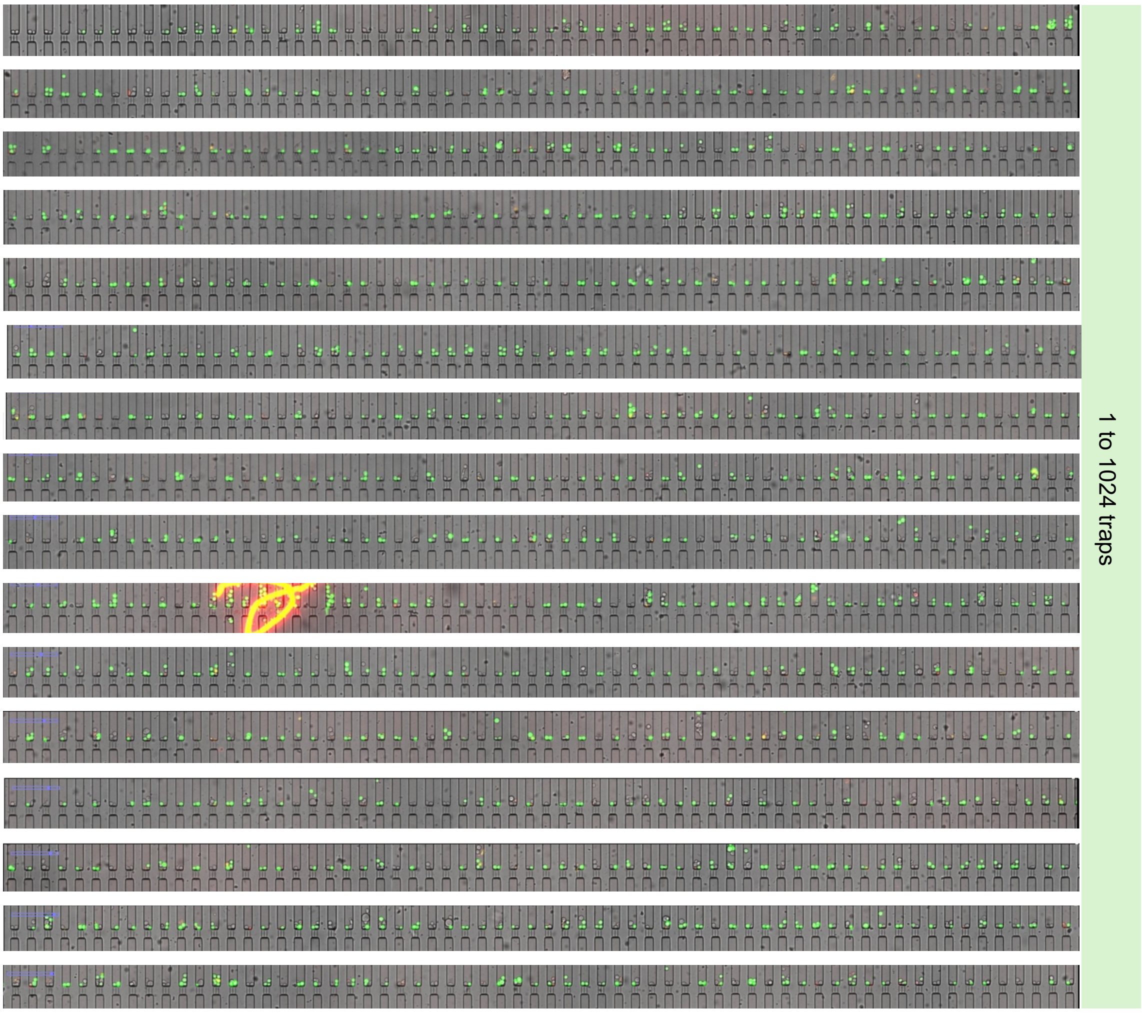

(continued…)

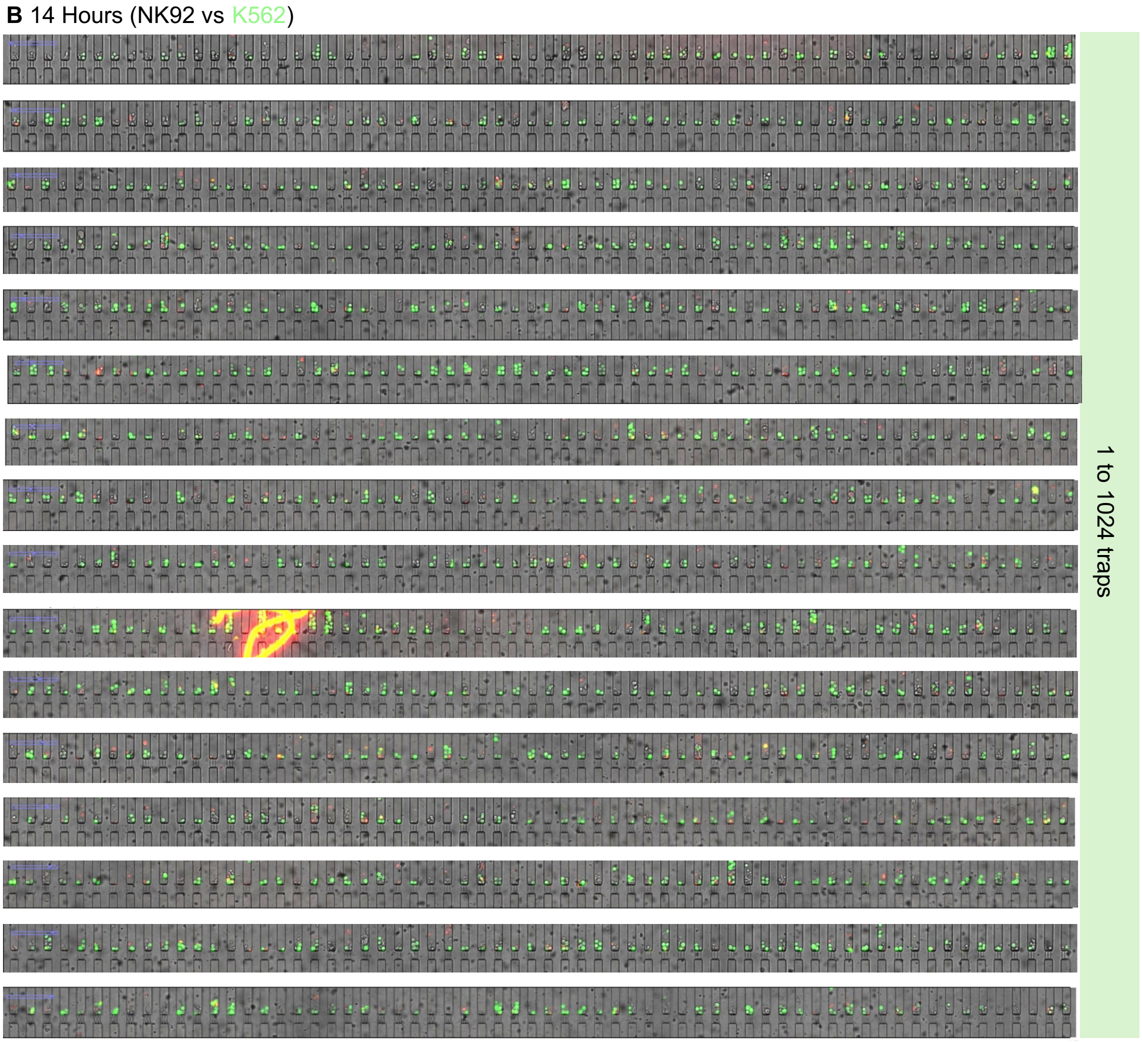

Figure S6. Array-wide visualization of cell distribution and retention. High-resolution stitched panoramic images displaying the entire 1,024-trap array at the initiation (t = 0 hours) and conclusion (t = 14 hours) of the cytotoxicity assay. NK92^{IL2} effectors and K562 targets (green) were pre-mixed and stochastically seeded. The macro-scale visualization confirms robust cell retention, stable confinement, and the diverse array of E:T ratios generated across the device over the 14-hour experimental window.

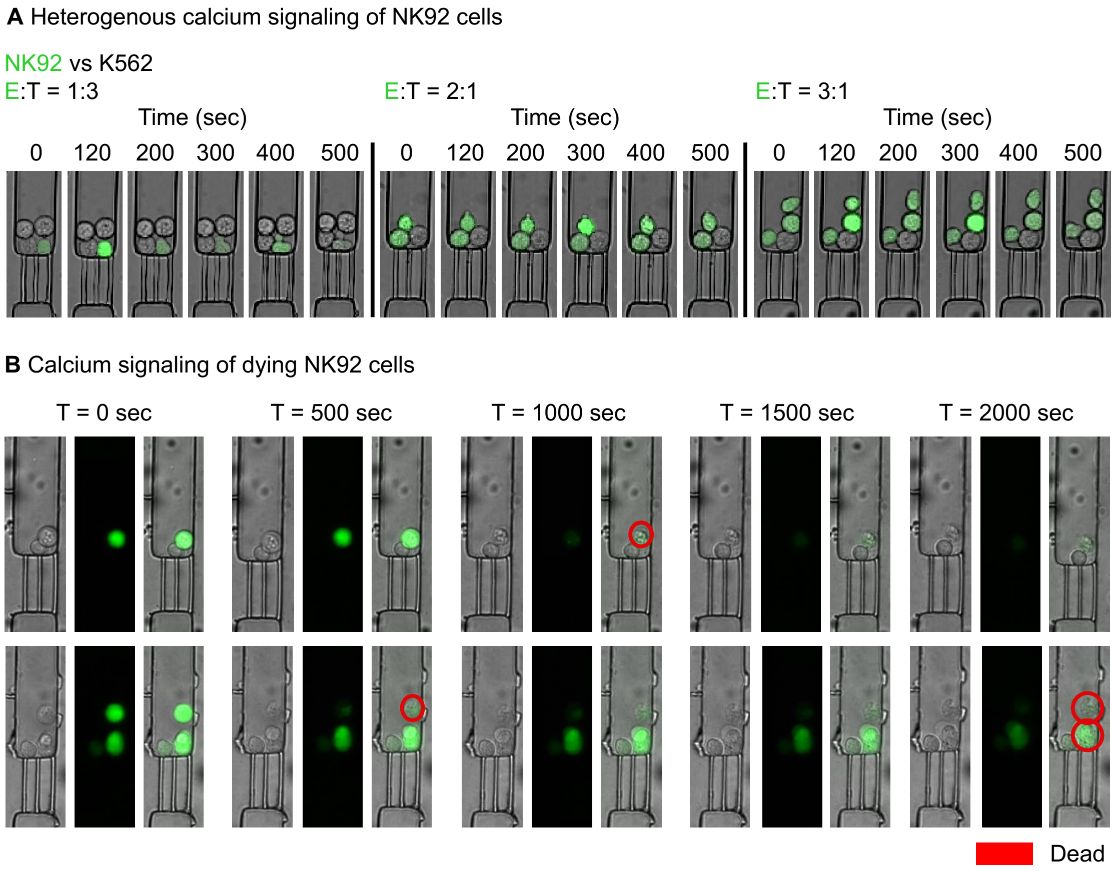

Figure S7. Heterogeneous calcium response and death signature of NK92 cells. (A) Immune cells exhibit a heterogeneous calcium response in the presence of target cells. In traps containing multiple NK92 cells (e.g., E:T = 2:1 or 3:1), individual effectors display distinct functional states; active calcium flux can be observed in one NK92 cell, while adjacent immune cells remain entirely non-responsive over the 500-second observation window. (B) In healthy effector cells, physiological calcium signaling typically presents as transient peaks over time. In contrast, cells undergoing death exhibit a sustained, stagnant high-intensity calcium signal for an extended period (up to 2000 seconds), followed by a complete loss of signal (highlighted with red circles). This prolonged elevation is attributed to the catastrophic disruption of intracellular calcium storage organelles and the loss of plasma membrane integrity.

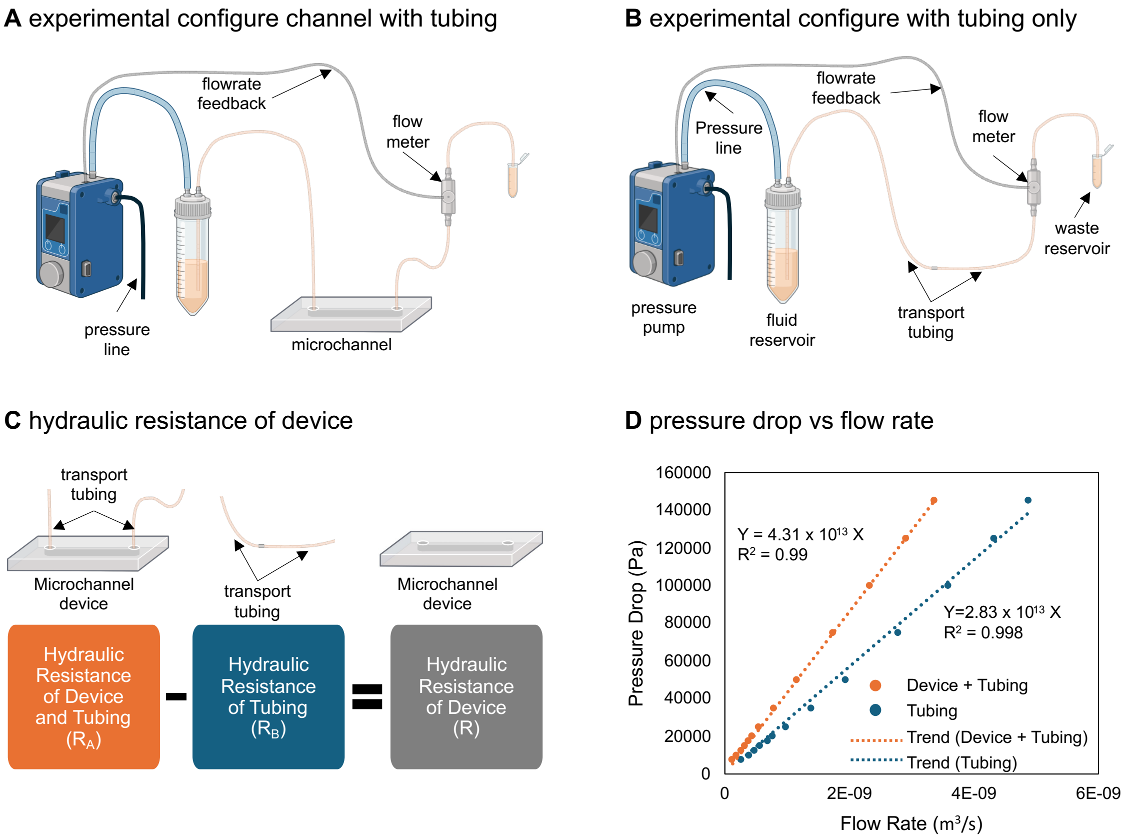

Figure S8. Evaluation of the hydraulic flow resistance of the CellTrap device. (A) An experimental configuration to estimate the hydraulic resistance of the device and connecting tubing for a given inlet pressure (*P*) by the pressure pump and measured outlet flow rate (*Q*) using the flow sensor. (B) An experimental configuration to estimate the hydraulic resistance of only the tubing, when the device is disconnected from the fluidic circuit, for a given inlet pressure (*P*) by the pressure pump, and measured outlet flow rate (*Q*) using the flow sensor. (C) The hydraulic resistance of the device is computed by subtracting the hydraulic resistance of the tubing from the combined hydraulic resistance of the device and tubing. (D) The inlet pressure (*P*) and the flow rate (*Q*) are plotted for configurations (A) and (B). The slopes of the trendlines are equivalent to the hydraulic resistances of the two configurations, i.e., $R_A = 4.31 \times 10^{13}$ Pa.s/m^3 and $R_B = 2.83 \times 10^{13}$ Pa.s/m^3. The hydraulic resistance of the CellTrap device $R = R_A - R_B = 1.48 \times 10^{13}$ Pa.s/m^3.

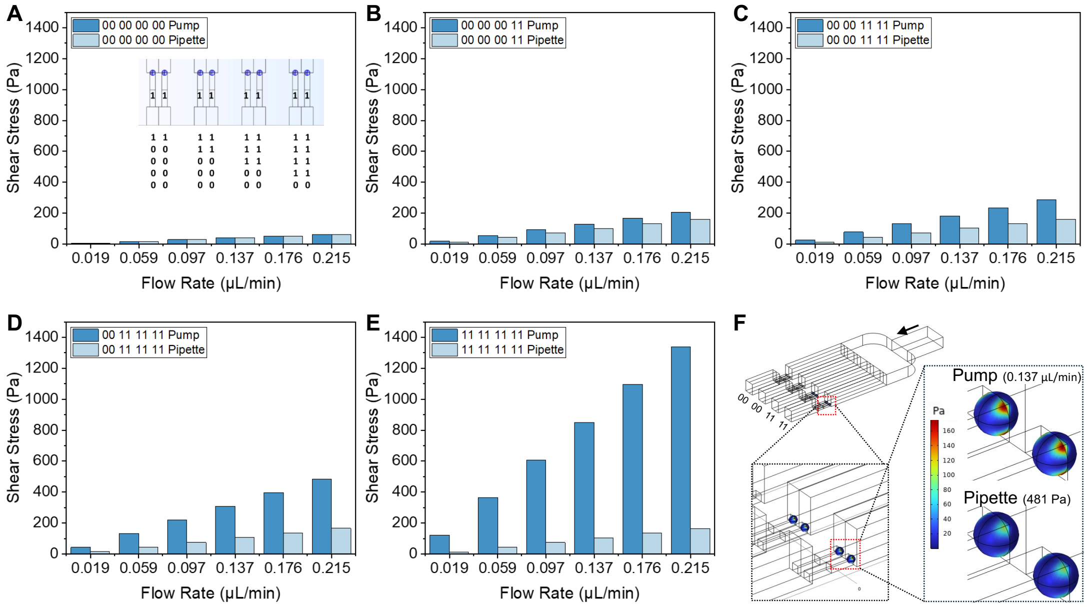

Figure S9. Numerical modelling of shear stress on trapped cells. (A-E) The COMSOL Multiphysics model is simulated to evaluate the maximum shear stress on trapped particles at various flow rates for both pump and pipette operations. Traps are considered under conditions of no obstruction and different particle occupancy. (F) COMSOL simulation representing shear stress at the same flow rate and pressure drop on particles obstructing the filter of traps.

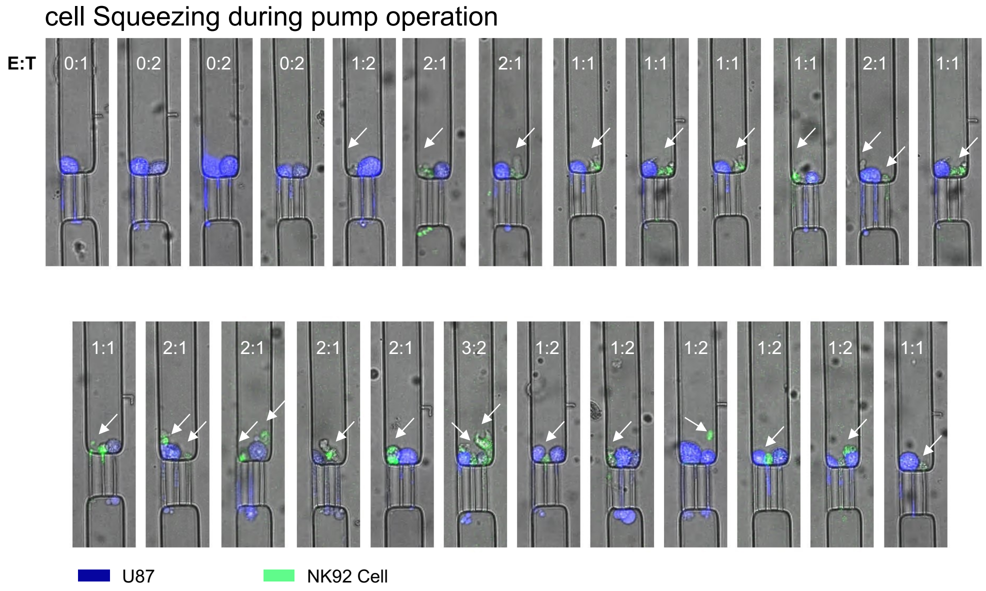

Figure S10. High shear effect on cells loaded using a syringe pump. U87 and $NK92^{IL2}$ cells are stuck in the filters when the sample is loaded using the pump. Therefore, the operation using a pipette is employed in the biological sample studies. White arrow representing $NK92^{IL2}$ cells.

Table S1. Comparison of existing cell interaction platforms and the CellTrap platform.

Platform Category	Control over E:T Ratios / Pairing	Equipment & Instrumentation Needs	Medium Exchange / Perfusion / Crosstalk	Device Complexity & Usability	Ref.
**1. Droplet microfluidics** (stochastic)	Stochastic (Poisson statistics)	**High** - typically requires precise pumps for continuous droplet generation and stable oil emulsion control	**No / None** - droplets are isolated, but continuous medium exchange is impossible during the assay	Complex setup; droplet stabilization is needed for long-term imaging, and downstream analysis can be challenging	1-5
**2. Droplet microfluidics** (deterministic; passive inertial ordering or active acoustic focusing)	**High** - improved control over one-to-one or defined pairing compared to stochastic droplet loading	**High** - requires precise multichannel flow control; passive ordering needs careful channel design, while acoustic focusing requires external actuation hardware	**No / None** - isolation is preserved, but sequential medium exchange remains difficult	Complex fluidic tuning; more controllable than stochastic droplets, but device operation and optimization are demanding	6-19
**3. Static droplets & sealed microwells** (stochastic; oil-sealed)	Stochastic (Poisson statistics)	**Low** - can often be pipette-loaded with minimal external instrumentation	**No / None** - oil sealing prevents cross-communication but simultaneously blocks medium exchange	Relatively simple to use, but oil handling can be inconvenient and long-term nutrient exchange is limited	20-23
**4. Array-based** (stochastic; 2D microwell or micropillar arrays)	Stochastic (Poisson statistics)	**Low to Moderate** - often relies on simple loading by sedimentation, pipetting, or basic flow	**Yes / Moderate to High** - shared bulk medium allows exchange, but neighboring compartments can easily communicate through diffusion	Simple planar design and easy imaging over large fields; however, it usually requires high cell concentrations and offers limited pairing precision	24-28
**5. Array-based cell traps** (deterministic - passive)	**High** - deterministic capture or positioning, often enabling one-to-one occupancy with well-designed geometries	**Moderate** - usually requires controlled flow (pumps) and highly optimized microstructures, but no external active field source	**Yes / Low to Moderate** - open/shared medium supports continuous perfusion and exchange, though isolation is less strict than in sealed droplets	Moderately complex; relies strongly on cell size or deformability, and device geometry must be carefully engineered to prevent clogging or escape	29-35
**6. Array-based cell traps** (deterministic - active)	**High** - strongest control over capture, positioning, and pairing using active external forces	**High** - requires electrical, magnetic, acoustic, or optical actuation systems alongside control hardware	**Yes / Low to Moderate** - continuous medium exchange is feasible, but compartment isolation depends heavily on the specific design	Most complex and expensive; requires specialized expertise, extra hardware, and often a larger physical footprint	18,36
**7. Our Platform (CellTrap)**	**Stochastic** (Poisson statistics)	**Minimal** - instrument-free; hydrostatic, pipette-driven loading	**Yes / Low** - isolated parallel flow paths allow continuous perfusion while minimizing diffusive communication	Simple, scalable device design; accommodates a broad range of cell sizes; easily enables sequential medium and reagent exchange on-chip	This work

Supporting references:

[1] S. Sarkar *et al.*, "Dynamic analysis of immune and cancer cell interactions at single cell level in microfluidic droplets," *Biomicrofluidics*, vol. 10, no. 5, Sep. 2016, doi: 10.1063/1.4964716.

[2] S. N. Agnihotri *et al.*, "Droplet microfluidics for functional temporal analysis and cell recovery on demand using microvalves: application in immunotherapies for cancer," *Lab Chip*, vol. 22, no. 17, pp. 3258–3267, 2022, doi: 10.1039/D2LC00435F.

[3] S. Sarkar *et al.*, "Machine learning-aided quantification of antibody-based cancer immunotherapy by natural killer cells in microfluidic droplets," *Lab Chip*, vol. 20, no. 13, pp. 2317–2327, 2020, doi: 10.1039/D0LC00158A.

[4] S. Antona, I. Platzman, and J. P. Spatz, "Droplet-Based Cytotoxicity Assay: Implementation of Time-Efficient Screening of Antitumor Activity of Natural Killer Cells," *ACS Omega*, vol. 5, no. 38, pp. 24674–24683, Sep. 2020, doi: 10.1021/acsomega.0c03264.

[5] Y. Yuan *et al.*, "Droplet encapsulation improves accuracy of immune cell cytokine capture assays," *Lab Chip*, vol. 20, no. 8, pp. 1513–1520, 2020, doi: 10.1039/C9LC01261C.

[6] M. Ali, W. Kim, M. S. Khan, M. A. Sahin, G. Destgeer, and J. Park, "Droplet acoustofluidics: Recent progress and challenges," *Biomicrofluidics*, vol. 19, no. 3, May 2025, doi: 10.1063/5.0261531.

[7] Y. Du, C. Wen, Y. Luo, and Y. Liu, "Deterministic Co-encapsulation of Microparticles in Droplets via Synchronized Merging for Single-Cell Genomics," *Anal. Chem.*, vol. 97, no. 47, pp. 26230–26236, Dec. 2025, doi: 10.1021/acs.analchem.5c05488.

[8] X. Luo and A. P. Lee, "Overcoming double Poisson limitation for co-encapsulation in droplets through hydrodynamic close packing of cells," *Microfluid. Nanofluidics*, vol. 27, no. 1, p. 3, Jan. 2023, doi: 10.1007/s10404-022-02600-9.

[9] M. Sesen and G. Whyte, "Image-Based Single Cell Sorting Automation in Droplet Microfluidics," *Sci. Rep.*, vol. 10, no. 1, p. 8736, May 2020, doi: 10.1038/s41598-020-65483-2.

[10] Q. Zhang *et al.*, "Development of a facile droplet-based single-cell isolation platform for cultivation and genomic analysis in microorganisms," *Sci. Rep.*, vol. 7, no. 1, p. 41192, Jan. 2017, doi: 10.1038/srep41192.

[11] L. Nan *et al.*, "Droplet-based single-cell pairing for high-throughput interaction mapping of antigen-receptor combinations," *Sci. Adv.*, vol. 11, no. 50, Dec. 2025, doi: 10.1126/sciadv.aeb1515.

[12] S. Hu *et al.*, "Large-Area Electronics-Enabled High-Resolution Digital Microfluidics for Parallel Single-Cell Manipulation," *Anal. Chem.*, vol. 95, no. 17, pp. 6905–6914, May 2023, doi: 10.1021/acs.analchem.3c00150.

[13] R. H. Cole *et al.*, "Printed droplet microfluidics for on demand dispensing of picoliter droplets and cells," *Proc. Natl. Acad. Sci.*, vol. 114, no. 33, pp. 8728–8733, Aug. 2017, doi: 10.1073/pnas.1704020114.

[14] P. Zhang *et al.*, "Deterministic droplet coding *via* acoustofluidics," *Lab Chip*, vol. 20, no. 23, pp. 4466–4473, 2020, doi: 10.1039/D0LC00538J.

[15] K. Shahrivar and F. Del Giudice, "Beating Poisson stochastic particle encapsulation in flow-focusing microfluidic devices using viscoelastic liquids," *Soft Matter*, vol. 18, no.

32, pp. 5928–5933, 2022, doi: 10.1039/D2SM00935H.

[16] T. Tang, H. Zhao, S. Shen, L. Yang, and C. T. Lim, "Enhancing single-cell encapsulation in droplet microfluidics with fine-tunable on-chip sample enrichment," *Microsystems Nanoeng.*, vol. 10, no. 1, p. 3, Jan. 2024, doi: 10.1038/s41378-023-00631-y.

[17] B.-U. Moon *et al.*, "Deterministic droplet-based co-encapsulation of single cells through inertial and hydrodynamic focusing," *Analyst*, 2026, doi: 10.1039/D5AN00986C.

[18] M. T. Chung, D. Núñez, D. Cai, and K. Kurabayashi, "Deterministic droplet-based co-encapsulation and pairing of microparticles via active sorting and downstream merging," *Lab Chip*, vol. 17, no. 21, pp. 3664–3671, 2017, doi: 10.1039/C7LC00745K.

[19] J. L. Madrigal *et al.*, "Characterizing cell interactions at scale with made-to-order droplet ensembles (MODEs)," *Proc. Natl. Acad. Sci.*, vol. 119, no. 5, Feb. 2022, doi: 10.1073/pnas.2110867119.

[20] S. H. Jin, H.-H. Jeong, B. Lee, S. S. Lee, and C.-S. Lee, "A programmable microfluidic static droplet array for droplet generation, transportation, fusion, storage, and retrieval," *Lab Chip*, vol. 15, no. 18, pp. 3677–3686, 2015, doi: 10.1039/C5LC00651A.

[21] L. Ding, P. Radfar, M. Rezaei, and M. E. Warkiani, "An easy-to-operate method for single-cell isolation and retrieval using a microfluidic static droplet array," *Microchim. Acta*, vol. 188, no. 8, p. 242, Aug. 2021, doi: 10.1007/s00604-021-04897-9.

[22] S. S. Bithi and S. A. Vanapalli, "Microfluidic cell isolation technology for drug testing of single tumor cells and their clusters," *Sci. Rep.*, vol. 7, no. 1, p. 41707, Feb. 2017, doi: 10.1038/srep41707.

[23] H.-H. Jeong, S. H. Jin, B. J. Lee, T. Kim, and C.-S. Lee, "Microfluidic static droplet array for analyzing microbial communication on a population gradient," *Lab Chip*, vol. 15, no. 3, pp. 889–899, 2015, doi: 10.1039/C4LC01097C.

[24] J. Wang, L. Du, Y. Han, D. Zhang, and D. Jing, "Advancing *in situ* single-cell microbiological analysis through a microwell droplet array with a gradual open sidewall," *Lab Chip*, vol. 23, no. 24, pp. 5165–5172, 2023, doi: 10.1039/D3LC00590A.

[25] Y. Xu, S. Zhou, Y. W. Lam, and S. W. Pang, "Dynamics of Natural Killer Cells Cytotoxicity in Microwell Arrays with Connecting Channels," *Front. Immunol.*, vol. 8, Aug. 2017, doi: 10.3389/fimmu.2017.00998.

[26] S.-E. Kim, S. Yun, and J. Doh, "Effects of extracellular adhesion molecules on immune cell mediated solid tumor cell killing," *Front. Immunol.*, vol. 13, Oct. 2022, doi: 10.3389/fimmu.2022.1004171.

[27] D. Moon, S.-E. Kim, C. Wang, K. Lee, and J. Doh, "Deep Learning-Based Automated Analysis of NK Cell Cytotoxicity in Single Cancer Cell Arrays," *BioChip J.*, vol. 18, no. 3, pp. 453–463, Sep. 2024, doi: 10.1007/s13206-024-00158-y.

[28] Y. J. Yamanaka, C. T. Berger, M. Sips, P. C. Cheney, G. Alter, and J. C. Love, "Single-cell analysis of the dynamics and functional outcomes of interactions between human natural killer cells and target cells," *Integr. Biol.*, vol. 4, no. 10, p. 1175, 2012, doi: 10.1039/c2ib20167d.

[29] F. A. Shaik *et al.*, "Pairing cells of different sizes in a microfluidic device for immunological synapse monitoring," *Lab Chip*, vol. 22, no. 5, pp. 908–920, 2022, doi: 10.1039/D1LC01156A.

[30] A. Desalvo, F. Bateman, E. James, H. Morgan, and T. Elliott, "Time-resolved microwell cell-pairing array reveals multiple T cell activation profiles," *Lab Chip*, vol. 20, no. 20, pp. 3772–3783, 2020, doi: 10.1039/D0LC00628A.

[31] K. Yin *et al.*, "Well-Paired-Seq: A Size-Exclusion and Locally Quasi-Static Hydrodynamic Microwell Chip for Single-Cell RNA-Seq," *Small Methods*, vol. 6, no. 7, Jul. 2022, doi: 10.1002/smtd.202200341.

[32] B. Dura, Y. Liu, and J. Voldman, "Deformability-based microfluidic cell pairing and fusion," *Lab Chip*, vol. 14, no. 15, p. 2783, 2014, doi: 10.1039/c4lc00303a.

[33] B. Dura, M. M. Servos, R. M. Barry, H. L. Ploegh, S. K. Dougan, and J. Voldman, "Longitudinal multiparameter assay of lymphocyte interactions from onset by microfluidic cell pairing and culture," *Proc. Natl. Acad. Sci.*, vol. 113, no. 26, Jun. 2016, doi: 10.1073/pnas.1515364113.

[34] Y. Zhou *et al.*, "Evaluation of Single-Cell Cytokine Secretion and Cell-Cell Interactions with a Hierarchical Loading Microwell Chip," *Cell Rep.*, vol. 31, no. 4, p. 107574, Apr. 2020, doi: 10.1016/j.celrep.2020.107574.

[35] G.-H. Lee, S.-H. Kim, A. Kang, S. Takayama, S.-H. Lee, and J. Y. Park, "Deformable L-shaped microwell array for trapping pairs of heterogeneous cells," *J. Micromechanics Microengineering*, vol. 25, no. 3, p. 035005, Mar. 2015, doi: 10.1088/0960-1317/25/3/035005.

[36] S. Ansaryan *et al.*, "High-throughput spatiotemporal monitoring of single-cell secretions via plasmonic microwell arrays," *Nat. Biomed. Eng.*, vol. 7, no. 7, pp. 943–958, Apr. 2023, doi: 10.1038/s41551-023-01017-1.

Table S2. Detailed number of combinations of empty, cancer control, immune control, singletons at different E:T, and multiples at different E:T for particles at $\lambda_1 = 0.51$, $\lambda_2 = 0.54$ and cells at $\lambda_1 = 0.83$, $\lambda_2 = 0.58$. Data associated with Figure 2.

	**Effector to Target** (E:T)	**Particles** ($\lambda_1 = 0.51$, $\lambda_2 = 0.54$)	**Cells** ($\lambda_1 = 0.83$, $\lambda_2 = 0.58$)
**Empty**	0:0	391	230
**Cancer control**	0:1	183	211
	0:2	49	92
	0:3	9	22
	0:4	0	4
**Immune control**	1:0	141	146
	2:0	52	57
	3:0	9	5
	4:0	0	0
**Singletons**	1:1	105	142
**Multiple**	2:2	8	11
	3:3	1	1
	4:4	0	0
	1:2	32	36
	1:3	5	3
	1:4	0	0
	2:1	26	42
	2:3	1	0
	2:4	0	0
	3:1	7	12
	3:2	4	5
	3:4	0	0
	4:1	1	4
	4:2	0	0
	4:3	0	1

Movie S1: Interaction of PBMCs with U87 cells at different effector-to-target (E:T) ratios. At an E:T ratio of 1:1, PBMCs actively engage with U87 cells, and after 5 hours, the membrane integrity of the U87 cells is lost in all five representative traps, indicating apoptosis. At an E:T ratio of 2:1, U87 cells show similar apoptotic behavior, and in addition, some PBMCs appear exhausted and dying in certain traps. At an E:T ratio of 1:2, we still observe the death of at least one U87 cancer cell. Finally, at an E:T ratio of 2:2, PBMCs can be seen killing both U87 cells. In the control group (E:T = 0:≥1), U87 cells remain viable throughout the entire imaging period.

Movie S2: Interaction of $NK92^{IL2}$ cells with U87 cells at different effector-to-target (E:T) ratios. At an E:T ratio of 1:1, $NK92^{IL2}$ and U87 cells interact within the channel, and the U87 cell can be seen dying within 12 hours. Over time, the $NK92^{IL2}$ cells also appear to become exhausted and die. At an E:T ratio of 2:1, the increased number of $NK92^{IL2}$ cells leads to U87 cell death within 8 hours. At an E:T ratio of 1:2, at least one U87 cell is killed by $NK92^{IL2}$ cells by the end of the 12-hour period. In the control group (E:T = 0:≥1), U87 cells remain viable throughout the entire observation period.

Movie S3: Calcium response of $NK92^{IL2}$ cells interacting with U87, LS174T, and K562 cells. The $NK92^{IL2}$ cells against U87 cells exhibit a gradual increase in calcium signal followed by a slow decline over a period of 980 seconds. The $NK92^{IL2}$ cells against LS174T cells show an initial rapid calcium peak followed by a decline over 980 seconds. Multiple subsequent peaks are observed, indicative of target recognition and repeated engagement. $NK92^{IL2}$ cells against K562 cells display a rapid initial calcium peak followed by a decline over 980 seconds, similar to their response against LS174T cells. Recurring peaks are observed, reflecting ongoing target identification and attack.
